# Visual attention, EEG alpha power and T7-Fz connectivity are implicated in prosthetic hand control and can be optimized through gaze training

**DOI:** 10.1186/s12984-019-0524-x

**Published:** 2019-04-27

**Authors:** J. V. V. Parr, S. J. Vine, M. R. Wilson, N. R. Harrison, G. Wood

**Affiliations:** 1 School of Health Sciences, Liverpool Hope University, Liverpool, UK; 2 College of Life & Environmental Sciences, University of Exeter, Exeter, UK; 3 Department of Psychology, Liverpool Hope University, Liverpool, UK; 4 Research Centre for Musculoskeletal Science and Sports Medicine Department of Sport and Exercise Science, Manchester Metropolitan University, Manchester, UK

**Keywords:** Myoelectric prosthesis, Amputees, Intervention, Conscious control, Therapy, Motor learning, Inter site phase clustering

## Abstract

**Background:**

Prosthetic hands impose a high cognitive burden on the user that often results in fatigue, frustration and prosthesis rejection. However, efforts to directly measure this burden are sparse and little is known about the mechanisms behind it. There is also a lack of evidence-based training interventions designed to improve prosthesis hand control and reduce the mental effort required to use them. In two experiments, we provide the first direct evaluation of this cognitive burden using measurements of EEG and eye-tracking (Experiment 1), and then explore how a novel visuomotor intervention (gaze training; GT) might alleviate it (Experiment 2).

**Methods:**

In Experiment 1, able-bodied participants (*n* = 20) lifted and moved a jar, first using their anatomical hand and then using a myoelectric prosthetic hand simulator. In experiment 2, a GT group (*n* = 12) and a movement training (MT) group (*n* = 12) trained with the prosthetic hand simulator over three one hour sessions in a picking up coins task, before returning for retention, delayed retention and transfer tests. The GT group received instruction regarding how to use their eyes effectively, while the MT group received movement-related instruction typical in rehabilitation.

**Results:**

Experiment 1 revealed that when using the prosthetic hand, participants performed worse, exhibited spatial and temporal disruptions to visual attention, and exhibited a global decrease in EEG alpha power (8-12 Hz), suggesting increased cognitive effort. Experiment 2 showed that GT was the more effective method for expediting prosthesis learning, optimising visual attention, and lowering conscious control – as indexed by reduced T7-Fz connectivity. Whilst the MT group improved performance, they did not reduce hand-focused visual attention and showed increased conscious movement control. The superior benefits of GT transferred to a more complex tea-making task.

**Conclusions:**

These experiments quantify the visual and cortical mechanisms relating to the cognitive burden experienced during prosthetic hand control. They also evidence the efficacy of a GT intervention that alleviated this burden and promoted better learning and transfer, compared to typical rehabilitation instructions. These findings have theoretical and practical implications for prosthesis rehabilitation, the development of emerging prosthesis technologies and for the general understanding of human-tool interactions.

**Electronic supplementary material:**

The online version of this article (10.1186/s12984-019-0524-x) contains supplementary material, which is available to authorized users.

## Background

Many upper-limb amputees rely on prosthetic hand devices to restore a degree of functionality to the performance of daily activities. Despite the increasing sophistication of these devices, they still provide less than 50% of the capability of an intact limb [1, 2], impose a high cognitive burden that results in fatigue and frustration [3], and are therefore frequently rejected [4]. The nature of this cognitive burden has recently been explored indirectly by examining disruption to visuomotor behaviours during prosthetic hand use [5, 6]. For example, Parr et al. [7] showed that when using a myoelectric prosthetic hand simulator, participants directed a greater amount of visual attention towards the prosthesis and objects being manipulated by it. This dependency on visual feedback to monitor and correct movements is in contrast to the feed-forward (target-focused) strategy revealed by skilled users in everyday tasks [8], and mirrors findings from novices in other domains (e.g. tool use [9] and laparoscopic surgery [10, 11]). Interestingly, it is this need to constantly, and consciously, pay close visual attention to movements that prosthesis users report as a key contributor to the cognitive burden experienced during prosthetic hand control [4, 12, 13]. The overall aim of this paper was to assess novel measures of this cognitive burden and to test the efficacy of a novel training technique that might reduce this burden.

Measures that directly evaluate this cognitive burden are needed in order to further our understanding of how efficient visuomotor behaviour is influenced by prosthesis use. Electroencephalography (EEG) is ideally suited for this purpose as it offers a window into the dynamics of ongoing neural activity with high temporal resolution. This is important, as the development of skilled motor performance is characterised by the precise allocation of processing resources to areas of the brain that are needed for successful task execution; termed ‘neural efficiency’ [14, 15]. It has been suggested that neural efficiency can be operationalised by cortical oscillations in the alpha frequency (8-12 Hz) [16]. Specifically, the magnitude (power) of alpha oscillations influence cortical activation by exerting inhibitory control and can therefore reveal a gating mechanism whereby resources are diverted away from regions showing higher alpha power (more inhibition) and towards regions showing lower alpha power (lower inhibition) [17]. Such a mechanism is reflected in evidence suggesting that during movement planning and execution, alpha power decreases over motor-related areas of the cortex while increasing over non-motor areas [18].

Using this gating model, research has shown that enhanced performance in motor tasks can be characterised by more efficient topographical alpha power distributions. For example, Gallicchio and colleagues have shown that lower central alpha power and higher temporal alpha power preceded improved performance in a biathlon shooting task [19] and were evident following a training period in golf-putting [20, 21]. Indeed, higher alpha power over the left-temporal region has been generally associated with improvements in motor learning and performance [22, 23], as conscious, verbal-analytical processes diminish as a function of automaticity and expertise [14, 24–27]. It is therefore plausible to assume that the cognitive burden experienced during initial prosthesis hand control is underpinned by both neural inefficiency, a dependence on vision to monitor hand state and that both may reflect a more conscious mode of prosthesis control.

## Experiment 1

The aim of the first experiment was to provide an evaluation of the cognitive burden experienced during initial prosthetic hand control in a visuomotor task, by simultaneously measuring visual attention and EEG alpha activity. By comparing task phases that require relatively low (Reach) and relatively high (Lift) levels of overt visual attention to the prosthetic hand [7], we also aimed to investigate the efficacy of inferring demands on cognitive processes from eye-movements alone. We hypothesised that when using a prosthesis simulator, participants would perform significantly slower compared to when completing the task using their anatomical hand. Second, and in line with Parr et al. [7], we hypothesised that this performance decrement would be underpinned by an increased dependence on vision, as indicated by increases in both hand-focused visual attention and time to shift gaze to the next target. Third, we hypothesised that prosthetic hand use would result in a global decrease in alpha power, reflecting increased cortical activation and more effortful performance [16, 22] – a decrease that should be more pronounced over the (left) temporal region of the brain [19]. Finally, we hypothesised that these disruptions would be greater for the more visually demanding ‘Lift’ phase compared to the less visually demanding ‘Reach’ phase.

## Methods

### Participants

Twenty right-handed participants (12 males and 8 females; age M = 25.32, SD = 5.05) volunteered for the study. Minimum sample size estimates were calculated using G*Power 3.1.9.2 [27] based on effect sizes reported in a previous expert-novice comparisons of cortical alpha activity [28]. To detect an effect size of ηp2 = .21 with an alpha of .05, a sample size of at least 18 was required to yield 80% power. Additional participants helped safeguard against possible data loss. Participants were able-bodied, had normal or corrected-to-normal vision and had no prior experience with a myoelectric prosthetic device. The study was approved by the local ethics committee and written informed consent was given prior to testing.

### Apparatus

#### Prosthesis

**Fig. 1:**
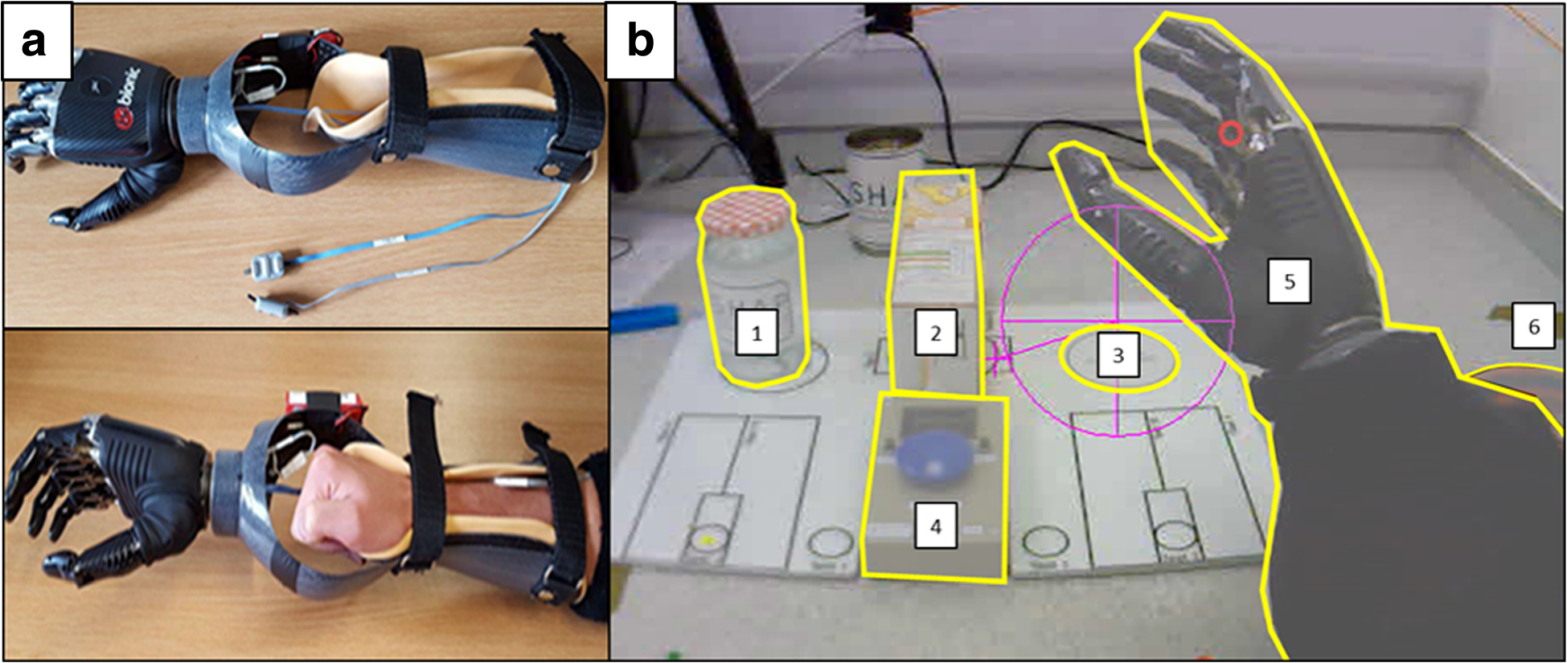
The myoelectric prosthetic hand simulator and the AOIs for the jar task. The prosthetic-hand simulator (**a**) and a screenshot taken from the Eyevision software (**b**). The screenshot shows the task environment and the 6 AOIs (1 = jar, 2 = carton, 3 = target, 4 = button, 5 = prosthesis, 6 = hand mat)

Participants wore the BebionicTM (Otto Bock HealthCare, Duderstadt, Germany) fully articulating myoelectric prosthetic hand simulator [7]. To fit able-bodied participants, the simulator was attached to the end of a carbon fibre trough in which the participants’ forearm and fist was positioned and fastened with Velcro straps (Fig. 1a). The prosthetic hand is controlled by muscle contraction detected by two electrodes placed on the extensor and flexor muscles in the forearm. These electrodes measure electrical changes on the skin covering the control muscles. Activation of the extensors triggered the opening of the hand whereas activation of the flexors triggered the closing of the hand.

#### The jar task

This task was taken from the Southampton Hand Assessment Procedure (SHAP) [29] which is a clinical tool used to measure hand dexterity. For this experiment, we chose the SHAP “lifting a heavy object” task. This required participants to lift a water-filled jar from the left side of the board over an empty carton and onto a designated area on the right side of the board as quickly and accurately as possible (Fig. 1b). Participants were required to begin each trial with their hand on a specified hand mat before (at a time of their own choosing) initiating the trial with the press of a button located centrally on the board. Following the successful placement of the jar, the task was terminated by a second button press.

#### Mobile eye-tracker

Gaze behaviour was measured with an Applied Science Laboratories (ASL; Bedford, MA) Mobile Eye XG gaze registration system that measures eye line of gaze at 30 Hz. Data were recorded directly onto a laptop (Dell Inspiron 6400) with ‘Eye-vision’ software installed. Video data from the eye-tracker were analysed offline using Quiet Eye Solutions software (Quiet Eye Solutions Inc.) which enables detailed frame-by-frame coding of the motor action and gaze behaviour of the performer. For each frame, gaze was manually determined to be lying within one area of interest (AOI) by the researcher, defined in Fig. 1. On occasions where two AOIs overlapped, priority was given to the AOI that was initially fixated upon so long as the obscuring AOI did not cause the position of this fixation to change. If gaze shifted from its position following AOI overlap then priority was given to the now obscuring AOI. Fixations made outside of AOIs were collectively labelled as “Other”. To understand the disruptions to gaze throughout the different phases of the task, the task was broken down into two distinct movement phases; reach for the jar (Reach), and lift the jar (Lift).

#### EEG

During the testing period, 64 active electrodes were positioned on the scalp according to the 10–20 system. Four additional electrodes were also placed above and below the left eye, and on the outer canthi of both eyes, to record the vertical electrooculogram (VEOG) and horizontal electrooculogram (HEOG). The signal was amplified and digitized at 512 Hz using the ActiveTwo recording system (Biosemi, the Netherlands). This system replaces the ground electrode used in conventional systems with common mode sense (CMS) and driven right leg (DRL) electrodes to enhance the common mode rejection ratio of the signal. Offline, signals were separately epoched from − 1250 ms to + 250 ms for the *Reach* phase and from − 250 ms to + 1250 ms for the *Lift* phase relative to the time the jar was first lifted from the table in each individual trial. We chose to segment signals this way as (a) it allowed a standardised movement phase across hand conditions despite differences in performance time and (b) it allowed an examination of two distinct movement phases that demand relatively low (the Reach phase) and high (Lift phase) dependence on vision [7]. This therefore offered the best opportunity to analyse the relationship between the dependence on vision and neural efficiency. The timing of these events was indicated via the recorded gaze videos derived from the eye-tracker, and were manually inputted into the EEG data as triggers offline following data collection.^1^ Signals were then band-pass filtered from 1 to 35 Hz (Finite Infinite Response), and referenced to the average of all scalp electrodes. Data were then subject to Independent Component Analysis (Runica Infomax algorithm [30],) to remove components accounting for blinks, eye movements, and other non-neural activity. At this stage, if epochs were deemed too noisy they were removed from further analysis. Although ICA was used for artefact rejection purposes, subsequent analyses were conducted on EEG channel data, as the most relevant literature within the psychomotor domain has tested the alpha-gating phenomenon via the mean regional activation occurring across selected EEG channels [19, 21, 31]. The spatial information of the processed epochs was then enhanced by surface Laplacian estimation that acts as a spatial filter of EEG potential distribution to reduce head volume conductor effects and eliminate electrode reference influence [32].

### Procedure

Upon arriving for testing, participants were informed of the purpose of the investigation and were sat comfortably on a chair so their elbows were in a 90 degree flexed position when resting on the table, as per SHAP instructions. They were then prepared for electrooculographic (EOG) and EEG measurements. The eye tracker was then fitted and calibrated by asking participants to direct their gaze to eight different points marked within the scene. Gaze behaviour was continuously monitored throughout testing and recalibrated at least every 15 trials, or when calibration had been lost. Participants first performed 30 trials of the task with their anatomic right hand before being introduced to the myoelectric prosthetic hand. This ensured that all prosthesis data reflected the difficulty in controlling the device rather than reflecting any deficit in understanding the task. Once fitted with the prosthetic hand simulator, participants were allowed to practice sending open and close signals. Once participants were able send five consecutive open and close signals, they were given one full practice trial before completing 30 full experimental trials.

### Measures

#### Performance

Performance was measured as the time (in seconds) taken to successfully complete the task, as indicated by the timer that was initiated and terminated by the performers first (before the trial started) and second button press (after the trial ended).

#### Target locking strategy (TLS)

Previous research has shown that more proficient visuomotor performance is indexed by a high TLS, with performers spending most of their time fixating the to-be-manipulated target, whereas, less proficient performance is indexed by a switching strategy, with performers shifting gaze between the hand/tool and the to-be-manipulated target [7, 33, 34]. TLS was computed by subtracting the percentage of time spent fixating the hand (either anatomic or prosthetic) from the time spent fixating the target (jar/target area). Positive scores reflect more time fixating relevant targets whereas negative scores reflect more time spent fixating on the hand. A score of ‘0’ reflects equal time spent fixating on the hand and targets and represents a ‘switching strategy’. A fixation towards the target object of a current movement phase was considered “target focused” but would become “hand focused” as soon as the hand grasped or manipulated it. For example, during the Reach phase, fixations towards the jar were considered ‘target focused’ but as soon as the hand grasped the jar fixations to the jar were then classified as ‘hand-focused’.

#### Gaze shifting

This was calculated as the time taken to shift visual attention towards the target of the next task phase following the completion of the previous phase. If gaze was shifted to the next target before completion of the previous task phase, then a negative time was recorded, indicating that gaze was ahead of the hand. A positive time reflected the extent to which the eye was behind the action of the hand, indicating a need to guide/monitor the hand. Gaze shifting was therefore measured for the time taken to shift gaze to the jar after having pressed the start button (Reach), and for the time to shift gaze to the target location after having first lifted the jar from the board (Lift). This measure has previously been shown to predict proficient prosthetic hand control with poorer performers slower to shift gaze to the next object in the task sequence [7].

#### EEG alpha power

Time-frequency decomposition was performed through short-time Fast Fourier Transform (FFT) on 9 overlapping segments (overlap of 87.5%), each of 500 ms duration and linearly spaced, with centre points ranging from − 1000 ms to 0 ms for *Reach* and from 0 ms to 1000 ms for *Lift*, relative to jar lift. Prior to FFT, data points within each segment were Hanning tapered and 0-padded to reach 2000 ms, providing complex-valued coefficients with a precision of 0.5 Hz for each channel and trial separately. Power was calculated for the entire alpha frequency band (8–12 Hz) as the squared amplitude of each signal, which was then averaged across the nine overlapping segments obtained for both the *Reach* and *Lift* phases. Seven regions of interest (ROI) were chosen for further analysis; left temporal (T7, TP7, FT7), left central (C1, C3, CP1, CP3), frontal (F1, F3, Fz, F2, F4), right central (C2, C4, CP2, CP4), right temporal (T8, TP8, FT8), parietal (P1, P3, Pz, P2, P4) and occipital (O1, Oz, O2). Power was averaged across these channels to yield values for each region. As no neutral baseline could be identified, non-normal distributions and inter-individual differences were dealt with by employing a median-scaled log transformation (see [19]. This transformation is implemented by scaling all power values for each participant (across all electrodes, trials, segments and conditions) by the median power value within that participant, before then employing a 10 log10 transformation to all values. EEG signals were processed using the EEGLAB toolbox [30] and custom MATLAB scripts (Mathworks, Natick, MA).

### Statistical analyses

#### Performance

A Shapiro-Wilk’s test revealed that performance data for the prosthesis condition were significantly non-normally distributed (*p* = .03). A Wilcoxon signed ranks test was therefore used to compare the time taken (in seconds) to complete the task between hand conditions.

#### TLS

To directly complement the EEG data, a 2 × 2 repeated measures analysis of variance (ANOVA) with the factors hand (anatomic, prosthetic) and phase (Reach, Lift) was performed on the TLS implemented by participants during the second prior to (Reach phase) and the second after (Lift phase) lifting the jar from its position.

#### Gaze shifting

For gaze shifting, a 2 (hand) × 2 (phase) repeated measures ANOVA was also conducted to compare the effect of hand condition on gaze shifting time for the Reach and Lift phases.

#### Alpha gating

A 2 × 2 × 7 repeated measures ANOVA with the factors hand, phase, and ROI (left temporal, left central, frontal, right central, right temporal, parietal, occipital) was performed on absolute alpha power to evaluate how the regional gating of alpha is altered across anatomic and prosthetic hand control. Furthermore, by comparing the Reach phase and the Lift phase, we evaluate how the extent of hand-related visual attention may influence the gating of alpha power.

Non-parametric effect sizes were calculated as, $r=Z/\sqrt{N}$[35], where *Z* is the test statistic and *N* is the total sample size. For all ANOVAs, Greenhouse-Geisser corrections were applied when sphericity was violated and effect sizes were calculated using partial eta squared (ηp2). All pairwise comparisons were adjusted via Bonferroni corrections to counteract the problem of multiple comparisons.

## Results

### Performance

Participants performed significantly slower during the prosthesis (Mdn = 6.35 s) compared to the anatomical (Mdn = 1.56 s) hand condition, Z = − 3.92, *p* < .001, r = − 0.87.

### TLS

**Fig. 2:**
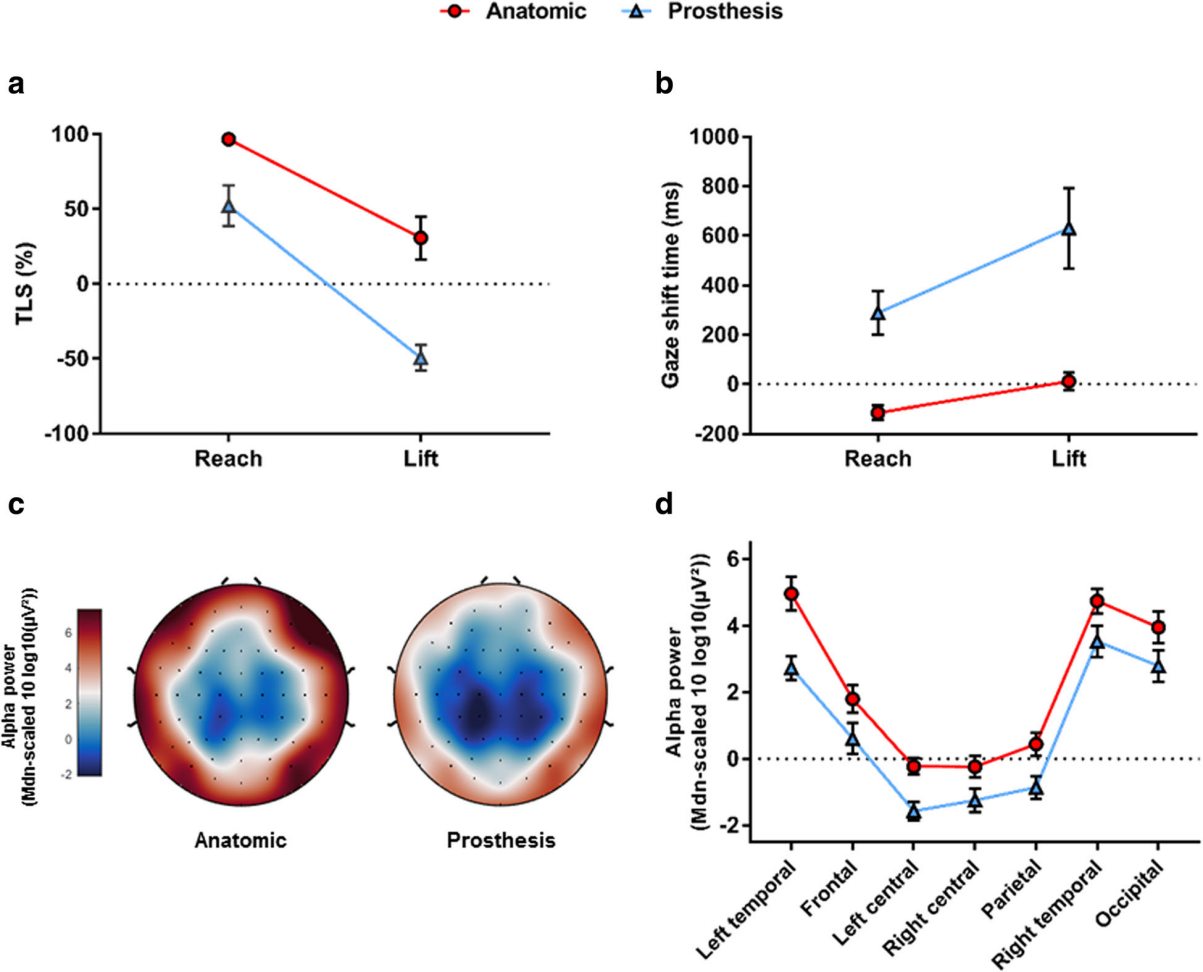
Gaze and EEG data for the jar task. Mean (± SD) target locking scores (**a**) for the anatomic and prosthetic hand simulator conditions across the two phases of the task. Positive scores reflects more time spent looking at targets and negative scores reflect more time looking at the hand. Mean (± SD) time in milliseconds to shift gaze (**b**) for the anatomic and prosthetic hand simulator conditions across the two movement phases. Positive times reflect a gaze shift after completion of a task phase whereas a negative time reflects a gaze shift prior to the completion of the task phase. Scalp topoplots (**c**) representing the global distribution of alpha power across hand conditions. Line plot (**d**) representing alpha power (± s.e.m) recorded from each region of interest (ROI) for both the anatomic and prosthetic hand condition. As there was no effect of task-phase presented values for both (**a**) and (**b**) represent the average of the two phases (reach and lift) for each ROI

There was a significant main effect of hand, *F* (1, 18) = 144.746, *p* < .001, ηp2 = 0.89, and phase, *F* (1, 18) = 255.904, *p* < .001, ηp2 = 0.93. There was also a significant hand x phase interaction, *F* (1, 18) = 30.562, p < .001, ηp2 = 0.63. Pairwise comparisons revealed that TLS was significantly lower during the prosthetic hand condition across both phases (*ps* < .01), and that for both anatomic and prosthetic hand conditions TLS was lowest during the Lift phase (*p* < .001; Fig. 2a).

### Gaze shifting

Results showed a significant main effect of hand, *F* (1, 19) = 269.974, *p* < .001, ηp2 = 0.934, phase, *F* (1, 19) = 129.360, p < .001, ηp2 = 0.872, and a significant hand x phase interaction, F (1, 19) = 15.746, *p* = .001, ηp2 = 0.453. Post hoc pairwise comparisons revealed that participants were significantly slower to shift their gaze when using the prosthesis across both phases (*p* < .001). They also revealed that participants were slowest to shift their gaze during the Lift phase for both hand conditions (*p* < .001; Fig. 2b).

### Alpha gating

Results revealed a significant main effect of hand, *F* (1, 19) = 28.942, *p* < .001, ɳp^2^ = .604, indicating a global decrease in alpha power that occurred during prosthetic hand use. Results also revealed a main effect of ROI, *F* (6, 114) = 52.044, *p* < .001, ɳp^2^ = .733, in which alpha power was lowest over the central and parietal regions, higher over the frontal region, and highest over the temporal and occipital regions for both anatomic and prosthetic hand control (Fig. 2c, d). There was no significant main effect of phase, *F* (1, 19) = 0.765, *p* = .393, ɳp^2^ = .039. No significant interactions were present (Fig. 2c, d).

## Discussion

This study provides the first direct examination of the cognitive burden associated with prosthetic hand control. As predicted, participants performed significantly (~ 4 times) slower when using the prosthesis simulator compared to their anatomical hand. Furthermore, this performance decrement was underpinned by spatial and temporal disruptions to hand-eye coordination. In line with Parr et al. [7], participants exhibited significantly lower TLS (more hand-focused gaze) and significant delays in the time to disengage from hand movements in all phases of the task. This again supports the idea that novice prosthetic hand use is reflected by an increased dependence on vision to monitor hand movements [5–7] and the inability to fixate targets ahead of time [7]. As hypothesised, the phase of the task that required the highest dependence on vision was the Lift phase [7]. During this phase, participants dedicated considerably more visual attention to the hand than the target (Mean TLS = − 49%) and took ~ 600 ms to disengage gaze from the jar following its pick up (the first 30% of the entire Lift phase).

When examining regional alpha power, our results revealed a focal pattern in which neural resources were directed away from occipital and temporal regions (generally highest alpha power) and diverted towards central and parietal regions (generally lowest alpha power), a pattern that was insensitive to both hand condition and movement phase. This pattern is in line with the gating-by-inhibition hypothesis [17] and supports research evidencing the bilateral activation of sensorimotor processes required to perform reaching and grasping movements [36]. It was surprising that this gating pattern was insensitive to hand condition given previous research has shown specific regional changes that occur as a function of expertise [14] and learning [31]. This is particularly the case for the left-temporal region that is thought to represent the conscious verbal processes present in the early stages of learning. However, such an effect may have been masked by the global decrease in alpha power that occurred during the prosthetic hand condition. Indeed, previous research has shown that novice performers exhibit a greater decrease in global alpha power compared to experts in visuomotor tasks [16, 25, 26], reflecting the increased cortical activation and mental effort required to perform the task [25]. Our results therefore support the hypothesis that initial prosthetic hand is underpinned by decreased neural efficiency as well as an increased dependence on vision. Examination of global alpha power could therefore provide a measure of skill development or cognitive effort to compliment measures of gaze in future studies.

However, contrary to our hypotheses, alpha power was consistent across both phases of our task despite these phases requiring distinctly target focused (Reach) and hand focused (Lift) visual strategies. This suggests that the cognitive processes behind visual attention are not straightforward, and raises questions concerning the validity of inferring the cognitive burden imposed during prosthetic hand control from overt visual attention alone [6]. It is also possible that alpha power may not be a suitable measure to detect more subtle changes in cognitive functioning that develop throughout a task. Indeed, the link between alpha power and neural efficiency in motor tasks has primarily been based on expert-novice differences [14, 15, 25]. Based on these considerations, regional alpha power may be more suited to reflect more radical or long-term changes in the functional architecture of the brain.

While these results are exciting, and could be used to quantify the usability and embodiment of prosthetic devices, questions remain concerning whether this cognitive burden can ever be alleviated, and, if so, which training interventions would be best suited to facilitate this process. Here, we have established that initial prosthetic hand control disrupts performance, increases the dependence on vision, and decreases neural efficiency. An interesting question going forward is whether training a prosthesis user to use their eyes more effectively would increase neural efficiency and facilitate the acquisition of prosthetic hand control. In the next experiment, we attempt to answer these questions by examining the impact of a gaze training (GT) intervention on measures of neural efficiency, conscious control and prosthetic hand learning.

### Experiment 2

While there are no evidence based guidelines for teaching prosthesis use, instructions are generally very explicit in nature, focusing the patient’s attention on limb movement [37]. Such instruction encourages the accrual of declarative knowledge and the conscious control of movement that can place high demands on attentional resources [38]. This type of movement control is indicative of the early stages of learning where cognitive demands are high, performance is error strewn and vision is the dominant sensory modality used to supervise on-going action [24]. In contrast, GT interventions use observational learning principles to guide novice performers to adopt eye-movement behaviours that are indicative of experts. Not only has GT been shown to expedite skill acquisition in novices learning surgical skills [11, 33, 39], in patients with movement coordination disorders [40–43] and in sports performers [44–46], but this learning has been found to be more implicit [34], and less cognitively demanding [39] when compared to technical instructions focused on limb movements. GT may therefore prove fruitful for prosthetic hand rehabilitation by lowering demands on visual attention and potentially reducing conscious cognitive control.

A method of measuring conscious control is through EEG connectivity; the phase synchrony or “co-activation” between two signals from the brain, with high connectivity reflecting functional communication and low connectivity reflecting regional independence [47]. Increased conscious movement control can be reflected by increased high-alpha (10–12 Hz) connectivity between the motor planning (Fz) and verbal-analytical (T7) regions of the brain [48]. For example, T7-Fz connectivity has been shown to reduce as a function of expertise [14, 20], and increase in individuals who are exposed to explicit rather than implicit training instructions [48, 49], whereas connectivity between motor planning (Fz) and visuo-spatial (T8) regions are not as susceptible to change [50]. Indeed, these disparate connectivity patterns have been shown in various skills, including surgery [49], postural control [51], rifle shooting [14], and golf putting [20, 50].

As well as providing a novel method of testing the efficacy of GT, EEG connectivity can allow further investigation into the relationship between visual attention and neural efficiency. Whilst topographical alpha power may reveal more long term changes in the functional architecture of the brain that arise via practice, evidence has shown T7-Fz connectivity to actively change in response to the ongoing context of practice; such as implicit vs explicit learning [49], internal vs external focus of attention [52], and increased task difficulty [51]. In fact, Ghasemian et al. [53] showed direct evidence that changes in EEG connectivity are sensitive to both short-term (same day) and long-term (1 week) training, whereas changes in EEG power are more affected by long-term changes. Therefore, alpha connectivity may be better suited to reflect a more immediate link between visually guided and consciously controlled movement than alpha power.

In this second experiment, we examined the efficacy of a GT intervention on prosthetic hand skill learning and retention compared to movement-related instructions typical of rehabilitation settings. Using a coin lifting task, we specifically focussed on the cortical dynamics occurring during object manipulation when demands on visual attention were highest. By doing so, we can clearly demonstrate how preventing learners from monitoring the prosthetic hand subsequently influences neural efficiency and learning. We also examined how effectively participants could transfer these skills to a more complex tea-making task. Accordingly, we make several hypotheses. First, we hypothesise that both interventions will facilitate performance improvements that should subsequently reduce the cognitive demands of the task. Second, we hypothesise that optimising gaze control (increased TLS & reduced gaze shifting) via GT will expedite learning and develop visuomotor strategies that are ultimately more neurally efficient (increased alpha) and less consciously controlled (reduced T7-Fz connectivity) compared to movement training (MT). As such, we expect a relationship between visual attention and conscious movement control to emerge. Finally, we hypothesise these benefits will be transferred to the more complex tea-making task.

## Method

### Participants

Twenty-four participants (12 male and 12 female, *M* = 24.36 years, *SD* = 7.23) participated in the experiment. Minimum sample size estimates were based on effect sizes reported in previous work showing the influence of explicit vs implicit learning on high alpha connectivity [49]. To detect an effect size of ηp^2^ = .285 with an alpha of .05, a sample size of at least 18 was required to yield 80% power. All participants were able-bodied, right-handed, had normal or corrected-to-normal vision, and had no prior experience with a prosthesis simulator. The study was approved by an institutional ethics committee and all participants provided written informed consent prior to testing.

### Apparatus

#### Prosthesis

The present study utilised the same myoelectric prosthesis used in Experiment 1.

#### Modified coin task

The task chosen for the present study was a modified version of the *picking up coins* task derived from the SHAP. This task was estimated to provide the best chance to examine a training effect, as Vasluain et al. [54] showed the number of participants failing to complete this task under the 35 s time limit reduced from 95 to 25% over the course of seven administrations of the entire SHAP protocol. This is in comparison to the much shorter and less complex jar task used in experiment 1, from which participants yielded an initial mean time of ~ 6 s. The task itself is made up of 4 *trial* coins placed approximately 30 cm away from the participant, 1 *start* coin positioned directly in front of the participant ready to grasp, and an empty glass jar in which all coins are to be placed. After successfully placing the *start* coin in the jar, participants were required to sequentially drag each *trial* coin to a desired *drag zone* so it could subsequently be placed into the jar. Each trial was completed following the placement of the final *trial* coin in the jar.

#### Tea-making transfer task

**Fig. 3:**
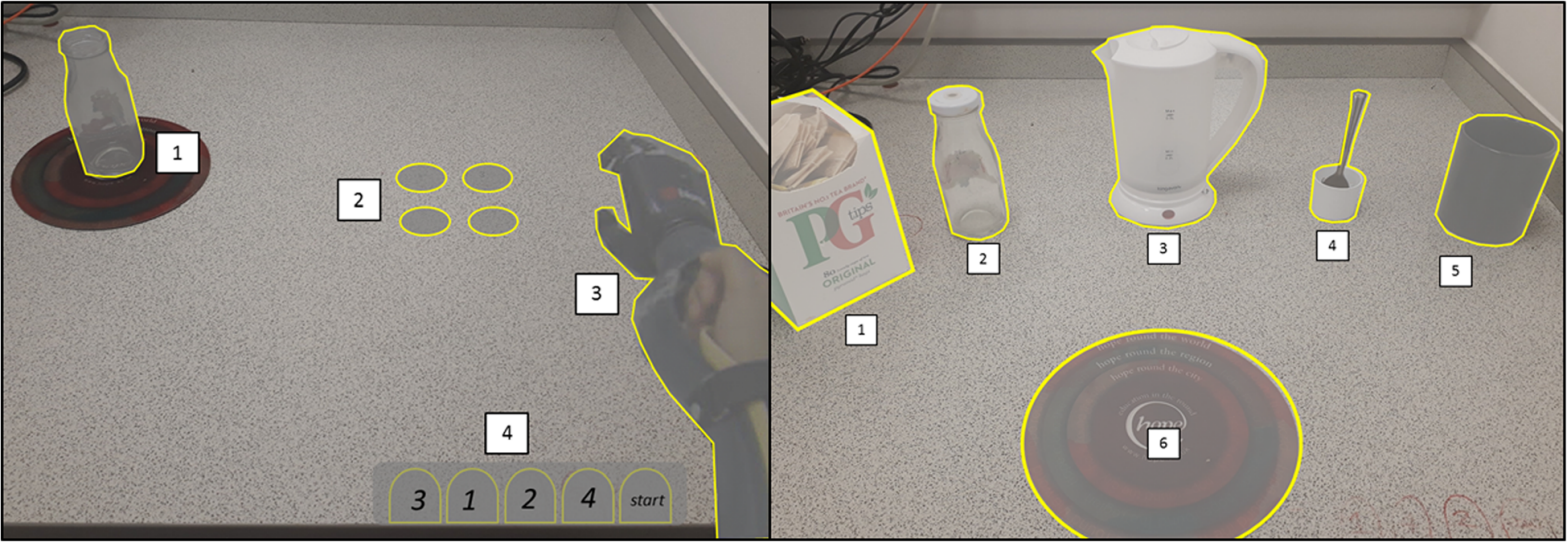
Experimental setup and AOIs for the coin task and the transfer tea-making task. The experimental set-up for our modified coin task (left), annotated with our four AOIs (1 = jar, 2 = coin (× 4), 3 = prosthesis, 4 = drag zone (× 4)). On the right is the experimental set-up for the transfer tea-making task, annotated with our 6 main AOIs (1 = teabags, 2 = milk, 3 = kettle, 4 = spoon, 5 = mug, 6 = place mat) that were further subdivided into a total of 17 AOIs outlined in Additional file 1

To examine the transfer of learning, we included a tea-making task. This task was chosen as it requires participants to apply acquired myocontrol skills to novel task variants such as object size, object weight, and grasping angle. It also provided a novel comparison with previous accounts of visuomotor control during tea-making in able-bodied individuals [8]. The task was made up of six objects; a mug, teaspoon, kettle (filled with 200 ml of water), sugar cube, milk jar with a screw-top lid (filled with 100 ml water) and teabag (Fig. 3). To complete the task, participants had to place the mug onto the place mat, add a teabag, a sugar cube, milk, water, and stir the contents twice with a spoon. Participants were told they could perform these tasks in an order of their choosing, as long as they started by placing the mug onto the place mat, completed all steps and ended by stirring the spoon.

#### Mobile eye-tracker

Gaze behaviour was measured and analysed using the same equipment and analysis steps as experiment 1. For the *modified coin task*, four AOIs (jar, coin, drop zone, prosthesis) and three task phases (reach, grasp, and lift) were identified. For the more complex *transfer tea task,* a total of 19 AOIs and 17 task phases were identified (a complete breakdown can be seen in Additional file 1).

#### EEG

EEG data from one participant was removed from analysis due to excessive noise during the baseline recording. All data collection and pre-processing steps were identical to experiment 1, except here we used an array of 32 electrodes. This decision was made to decrease preparation time and data storage size to compensate for the increase in recording blocks. For the *modified coin task,* offline signals were specifically epoched to represent a *Lift* task phase. To do so, data were epoched from − 1250 ms to + 250 ms relative to the instance the coins made contact with the bottom of the jar following placement. This instance was detected using a custom-made microphone placed behind the jar that automatically inserted digital triggers into the EEG recording when it detected sound > 70 dB. Time-frequency decomposition was performed through short-time FFT on 9 overlapping segments (overlap of 87.5%), each of 500 ms duration and linearly spaced with centre points ranging from − 1000 ms to 0 ms. For the *tea-making* task, manually inserted triggers were linearly spaced every 500 ms between the start and end of each trial – identified via previous calibration. EEG data were then epoched from − 2000 ms to 0 ms relative to each trigger, resulting in 75% overlap to increase the signal to noise ratio during signal processing. Short-time FFT was performed on 17 overlapping segments (overlap of 87.5%), each of the duration of 500 ms and linearly spaced with centre points ranging from − 1750 ms to − 250 ms. Prior to FFT, data points within all segments (across both tasks) were Hanning tapered and 0-padded to reach 2 s.

#### Procedure

Participants were required to attend the laboratory on five consecutive days and a further day approximately one week later (*M* = 6.52 days, *SD* = 2.11) for a delayed retention and transfer test. On day 1, the experiment was explained, and participants were fitted with the EEG and eye-tracking equipment. Once participants were fitted with the prosthesis, and could demonstrate adequate control, the *coin task* was explained and a demonstration was given by the researcher using the anatomic limb and via a video demonstration showing the task performed with the prosthesis. Participants were then given one full practice trial (5 coins) before completing 15 consecutive experimental trials (75 coins).

Participants were randomly allocated into GT and MT groups, with sex differences equally distributed. The training period lasted from days 2 (T1) to 4 (T3) and required participants to perform 15 trials of the coin task on each visit. On day 2, the GT group was first shown a video derived from the eye-tracker that depicted a performer purposely adopting expert visual control whilst performing the task using the prosthesis. Audio commentary that overlaid the video highlighted the performer’s target-focused gaze strategy, and the speed at which gaze was shifted to target locations following the completion of each task phase [40–42]. Participants were then fitted with the eye-tracker and advised to mimic the gaze strategy of our *expert* in the 15 subsequent attempts that followed. Eye movements were again recorded on days 2 and 3 so participants could assess their attempts to mimic the expert model upon repeated viewing on days 3 and 4 [39].

For the MT group, a video of the same *expert* trial was shown on day 2 but from a third person perspective. This was done so participants could more easily be made aware of the smooth and direct manner in which the expert controlled the prosthesis – as was emphasised by the audio commentary [40–42]. The video also gave participants a set of movement rules to help describe the expert’s performance, such as “drag the coin with the tip of the thumb” and “position the thumb beneath the coin before grasping”. Like the GT group, participants were then advised to mimic the movement style of the expert in the 15 subsequent experimental trials. No eye-tracker was worn, instead participants were recorded (Finepix S6500fd) from the same third person perspective as their training video so participants could assess their attempts to mimic the movements of the expert model on days 3 and 4 [39]. For both groups, EEG was not recorded throughout training.

On day 5, no further instructions were given and participants were asked to perform a further 15 trails of the coin task whilst measures of EEG and eye-tracking were taken (i.e. non-delayed retention test). Before performing the tea-making transfer task, participants were provided with a demonstration by the researcher using the anatomic limb, and shown a video demonstration of the researcher performing a single trial using the prosthesis. Participants then repeated this procedure approximately 1 week later for delayed retention and transfer tests.

### Measures

#### Performance time

For the coin task, performance time was measured as the time (in seconds) elapsed between the successful placement of the *start* coin and the final *trial* coin into the jar, recorded by the researcher using a stopwatch (Casio, Japan). If a coin was dropped, time was continued as participants were instructed to move on to the next coin in the sequence whilst the researcher replaced the dropped coin. In the instance that a participant dropped the final coin, time was paused until the researcher replaced the coin upon its starting position. For the tea-making task, performance time was measured as the time elapsed (in seconds) between first grasping the mug and replacing the spoon following two stirs.

#### Performance error (coin drops)

To provide an indication of performance error within the coin task, we recorded the total number of coins that were dropped within each block of 15 trials.

### Visual attention

#### Target locking strategy (TLS)

TLS was measured following the same procedure as experiment 1.

#### Gaze shifting

Gaze shifting was also measured following the same procedure as experiment 1. However, for the tea-making task, gaze-shifting time was only recorded for the phases of the task that started with an object manipulation. This ensured the ensuing shift location (usually a drop location) was consistent across participants for each chosen phase, and did not reflect a more indecisive visual search behaviour that occurred when participants were in-between task phases.

### EEG

#### Alpha power

As the *actual* alpha frequency band can show inter-subject variability, standardising alpha (8–12 Hz) across all participants might prevent the detection of more subtle changes in alpha activity. The individual alpha frequency (IAF) of each participant was therefore detected using the eyes-closed centre of gravity method [55] to enhance the ability to detect the differential effects of training instruction. Power (μV^2^) was then averaged across overlapping FFT segments in the adjusted alpha frequency band (IAF-2 to IAF + 2) for each channel and trial. Based on previous research [31] seven regions of interest (ROI) were chosen; left temporal (T7, FC5, CP5), left central (C3, FC1, CP1), frontal (F3, Fz, F4), right central (C4, FC2, CP2), right temporal (T8, FC6, CP6), parietal (P3, Pz, P4) and occipital (O1, Oz, O2). Power was averaged across these channels to yield values for each region following median-log scaling [19, 31].

#### High alpha connectivity

Functional connectivity was computed as the intersite phase clustering (ISPC) over time. ISPC measures the phase lag consistency across time between two channels independently from their power and reflects functional connectivity between the oscillatory activity of two underlying cortical regions, with values ranging from 0 (no connectivity) to 1 (perfect connectivity). For both the coin task and the tea task, ISPC was calculated for each epoch using bespoke Matlab scripts as, $ISPC(f)=\mid {n}^{-1}\sum \limits_{w=1}^n{e}^{i\left(\theta x\left(w,f\right)-\theta y\left(w,f\right)\right)}\mid$, where *i* is the imaginary operator; *θx* and *θy* are the phase angles of the recorded signal at two different scalp locations at FFT time window *w* and frequency *f*; *e*^*i*(*θx*(*w*, *f*) − *θy*(*w*, *f*))^ denotes a complex vector with magnitude 1 and angle *θx* − *θy*; ${n}^{-1}\sum \limits_{w=1}^n\left(\cdotp \right)$ denotes averaging across the overlapping FFT time windows; and ∣ · ∣ is the module of the average vector [32]. ISPC values were then averaged over trials before being Fisher *Z* transformed (inverse hypabolic tangent), meaning values could range from 0 to ∞. Values were then averaged across channel pairs and the high-alpha frequency band (IAF to IAF + 2 Hz). In line with previous research, we focused on left temporal frontal (T7-Fz) and right temporal frontal (T8-Fz) connectivity.

### Data analyses

#### Performance

For the coin task, performance time and error were subject to a 2 × 6 mixed-design ANOVAs, with group (movement trained, gaze trained) as the between-subjects factor and time (baseline, T1, T2, T3, retention, delayed retention) as the within-subjects factor. For the tea-making transfer task, a Kruskal-Wallis tests was run to compare performance time between groups at retention and delayed retention due to violations of Shapiro Wilk’s test of normality. Within group changes from retention to delayed retention were then analysed using Wilcoxon signed ranks tests.

#### Visual attention

To align with EEG data, only TLS and gaze shifting data specific to the *Lift* phase were included for the coin task. For the tea-task, both measures were averaged over task phases to derive an overall indication of visual control. Both measures were then subject to a 2 (group) × 3 (time; baseline, retention, delayed retention) mixed-design ANOVA for the coin task, and a 2 (group) × 2 (time; retention, delayed retention) mixed design ANOVA for the tea-making task.

#### Alpha power

For the coin task, changes in regional alpha power were examined using a 2 (group) × 3 (time) × 7 (ROI) mixed design ANOVA. For the tea-making task, a 2 (group) × 2 (time) × 7 (ROI) mixed-design ANOVA was performed.

#### High alpha connectivity

For the coin task, changes in T7-Fz and T8-Fz connectivity over time were examined using a 2 (group) × 2 (hemisphere) × 3 (time) mixed-design ANOVA. To provide direct between group comparisons unbiased from baseline levels of connectivity, we also examined the change (∆) in ISPC values from baseline to retention (Ret ∆) and from baseline to delayed retention (Del ∆) using a 2 (group) × 2 (hemisphere) × 2 (time) mixed design ANOVA. Finally, baseline ISPC values derived from the coin task were also used to allow the same between group ∆ ISPC comparisons in the transfer tea-making task at retention and delayed retention.

#### Regression analyses

To directly explore the relationship between visual attention and conscious control, regression analyses were performed to determine if T7-Fz could be predicted using our measures of visual attention (TLS & gaze shifting) for our coin task.

## Results

### Coin task

#### Performance

For performance time, results revealed a significant main effect of time, *F* (3.08, 67.712) = 48.19, *p* < .001, η_p_^2^ = .687, a significant main effect of group, *F* (1, 22) = 6.94, *p* = .015, η_p_^2^ = .712, but no time x group interaction, *F* (5, 110) = 0.772, *p* = .572, η_p_^2^ = .034. Pairwise comparisons showed that the MT group performed significantly faster at T3 compared to B1 (*p* < .001) and T1 (*p* = .020), after which no further improvements were made (*p* = 1.00). Similar results were found for the GT group, who performed faster at T3 compared to B1 (*p* < .001), T1 (*p* = .001), and T2 (*p* = .091), but subsequently plateaued at retention and delayed retention (*p* = 1.00). Importantly, comparisons also revealed that whilst there were no significant difference between groups at B1 (*p* = .638) and T1 (*p* = .108), the GT group performed significantly faster than the MT group on all subsequent visits (*ps* = .022).

For performance error, results failed to reveal a significant main effect of time, *F* (5, 110) = 2.101, *p* = .071, η_p_^2^ = .087, suggesting the number of coin drops to be fairly insensitive to practice. There was also no main effect of group, *F* (1, 22) = 0.481, *p* = .495, η_p_^2^ = .021, and no time x group interaction, *F* (5, 110) = 0.745, *p* = .592, η_p_^2^ = .033.

#### Target locking score (TLS)

Results revealed a significant main effect of time, *F* (1.56, 34.24) = 9.97, *p* < .001, η_p_^2^ = .312, a main effect of group, *F* (1, 22) = 35.212, *p* < .001, η_p_^2^ = .410, and a significant time x group interaction, *F* (2, 44) = 13.481, *p* < .001, η_p_^2^ = .380. Post-hoc pairwise comparisons revealed no difference between groups at baseline (*p* = .686), but the GT group to exhibit significantly higher TLS compared to the MT group at retention and delayed retention (*p* < .001). Participants in the MT group showed no significant improvement from baseline to retention (*p* = 1.00) or baseline to delayed retention (*p* = 1.00). Conversely, the GT group significantly increased their TLS from baseline to retention (*p* < .001) and delayed retention (*p* < .001).

#### Gaze shifting

**Fig. 4:**
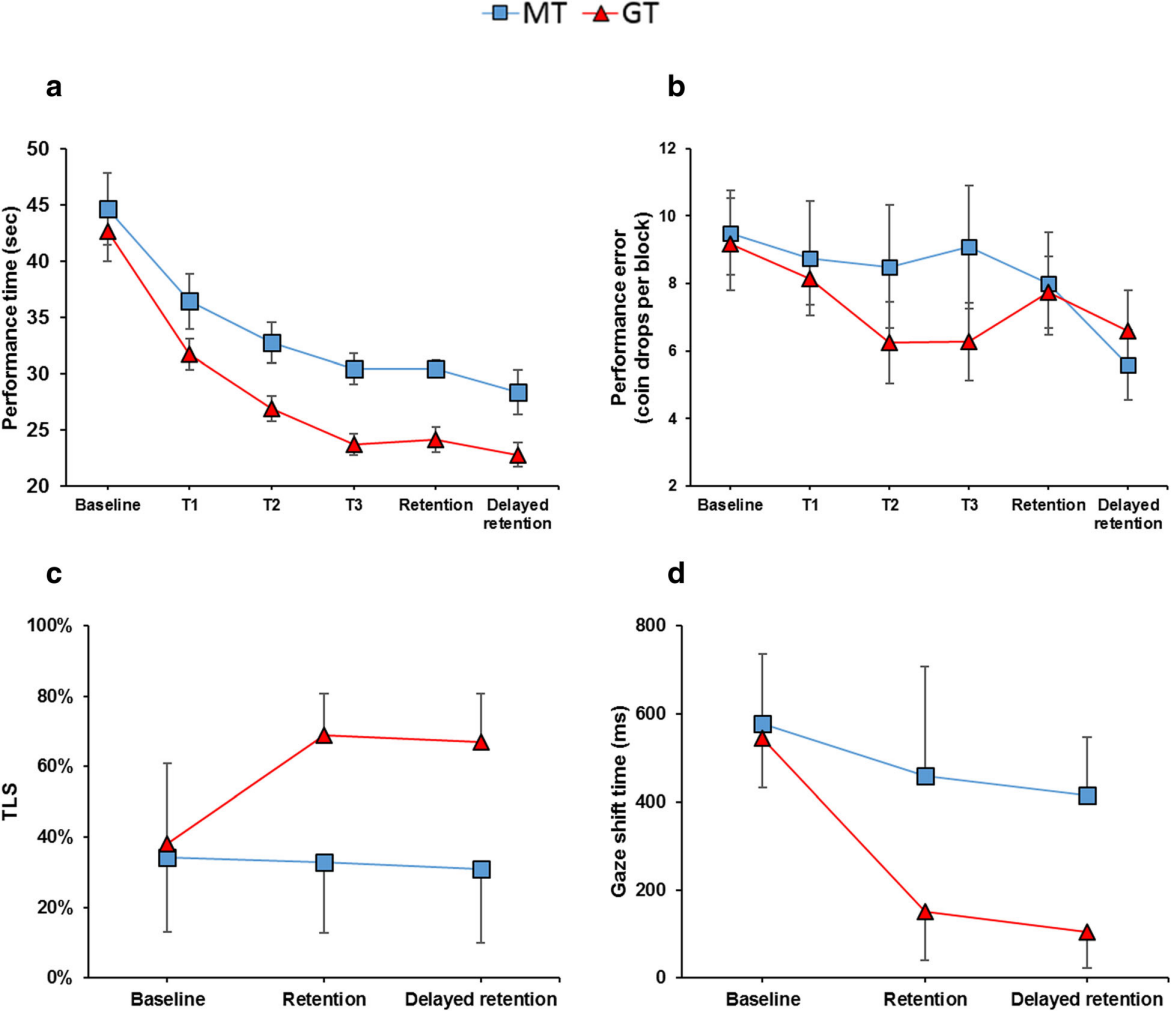
Performance and gaze data before, during and after training Line plots representing mean (± s.e.m) performance time (**a**) and performance error (**b**) in the coin task for both groups across time and the mean (± SD) target locking scores (**c**) and gaze shifting times (**d**) at baseline, retention and delated retention specific to the Lift phase of the task

Results revealed a significant main effect of time, *F* (1.29, 28.42) = 34.269, *p* < .001, η_p_^2^ = .609, a main effect of group, *F* (1, 22) = 26.902, *p* < .001, η_p_^2^ = .550, and a significant time x group interaction, *F* (2, 44) = 8.361, *p* = .001, η_p_^2^ = .279. Post-hoc pairwise comparisons revealed no difference between groups at baseline (*p* = .586), but the GT group to exhibit significantly faster gaze shifts than the MT group at retention (*p* = .001) and delayed retention (*p* < .001). They also revealed both the MT group (*ps* = .018) and the GT group (*p* < .001) shifted their gaze significantly faster from baseline to retention and delayed retention. Performance data and gaze data can be seen in Fig. 4.

#### Alpha power

**Fig. 5:**
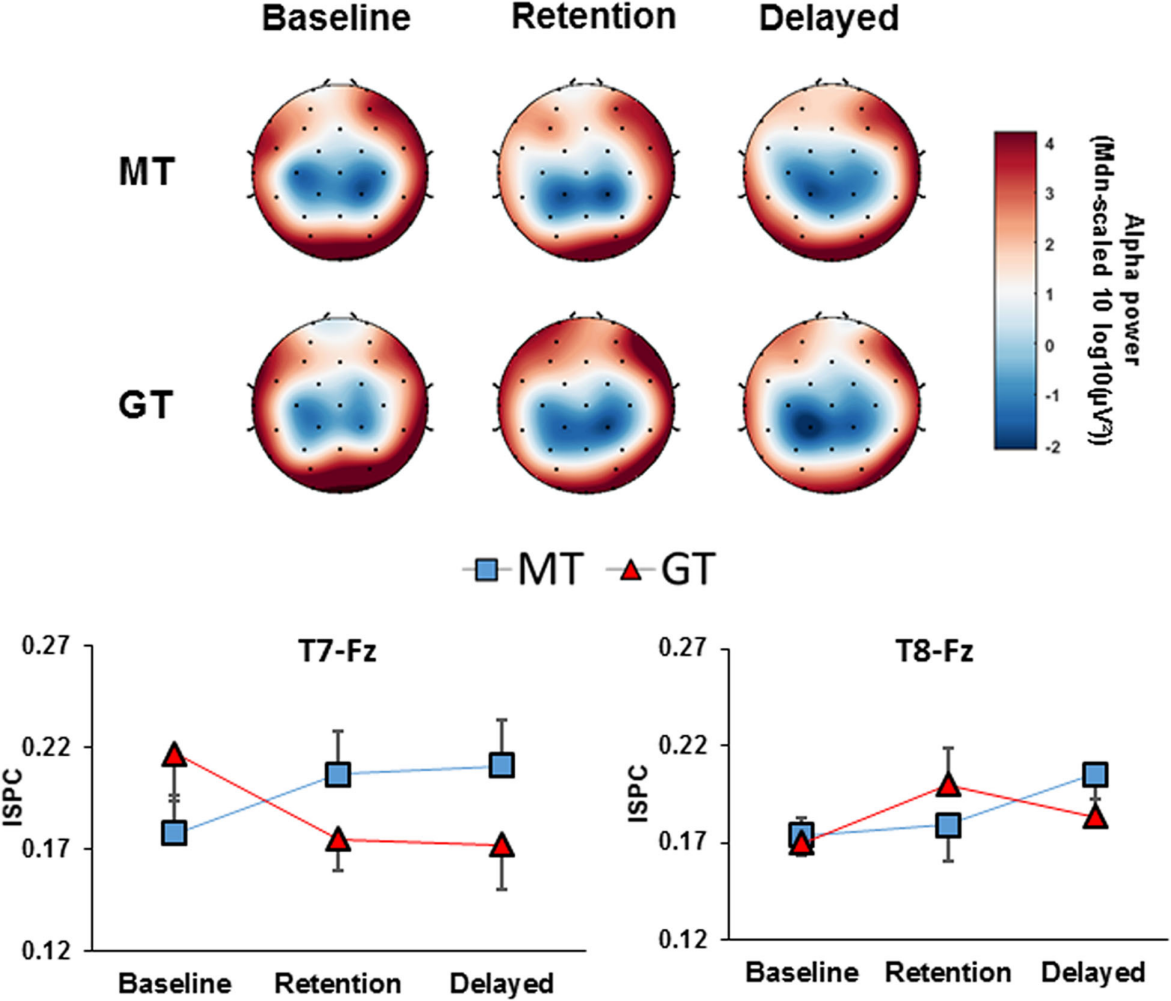
EEG data before, during and after training Scalp topoplots (top) representing the global distribution of alpha power for each group across the three time points. Displayed below are line plots representing the mean high-alpha inter site clustering (± s.e.m) between T7-Fz (left) and T8-Fz (right) for the MT and GT groups across time

For the coin task, the ANOVA also showed a significant main effect of ROI, *F* (3.712, 70.530) = 87.703, *p* < .001, η_p_^2^ = .822, revealing a focal pattern in which alpha was lowest over central and parietal regions, higher over temporal and frontal regions, and highest over the occipital region. There was also a significant main effect of time, *F* (2, 40) = 3.279, *p* = .049, η_p_^2^ = .049, and a significant time x ROI interaction, *F* (6.685, 127.022) = 2.819, *p* = .010, η_p_^2^ = .129. Pairwise comparisons revealed that both groups exhibited a significant decrease over the left-temporal (*p* = .001) and right temporal (*p* = .042) regions from baseline to delayed retention. All other interactions were non-significant (Fig. 5).

#### High alpha connectivity

When examining cross hemispheric (T7 vs T8) changes in temporal-frontal (Fz) connectivity, results showed no overall main effect of time, *F* (2, 40) = 0.427, *p* = .655, η_p_^2^ = .021, and no overall main effect of group, *F* (1, 20) = 0.156, *p* = .697, η_p_^2^ = .008. There was however a significant time x group interaction, *F* (2, 40) = 3.387, *p* = .044, η_p_^2^ = .145, and a significant time x hemisphere x group interaction, *F* (2, 40) = 4.532, *p* = .017, η_p_^2^ = .185. Pairwise comparisons revealed that participants in the GT group exhibited a significant reduction in T7-Fz connectivity from baseline to delayed retention (*p* = .043), and a marginally significant reduction from baseline to retention (*p* = .056). No changes were observed in the MT group (Fig. 5).

#### Δ high alpha connectivity

Results from ANOVA showed no effect of time, *F* (1, 20) = 0.260, *p* = .616, η_p_^2^ = .013, hemisphere, *F* (1, 20) = 3.333, *p* = .083, η_p_^2^ = .143, or group, *F* (1, 20) = 4.284, *p* = .052, η_p_^2^ = .176. There was however a significant hemisphere x group interaction, *F* (1, 20) = 7.934, *p* = .011, η_p_^2^ = .284, in which a significant difference between groups was observed only for the change in T7-Fz connectivity (*p* = .003). Pairwise comparisons also showed an overall significant difference between hemispheric changes for the GT group (*p* = .003) which consisted of a decrease in T7-Fz connectivity and an increase in T8-Fz connectivity.

#### Regression analyses

**Fig. 6:**
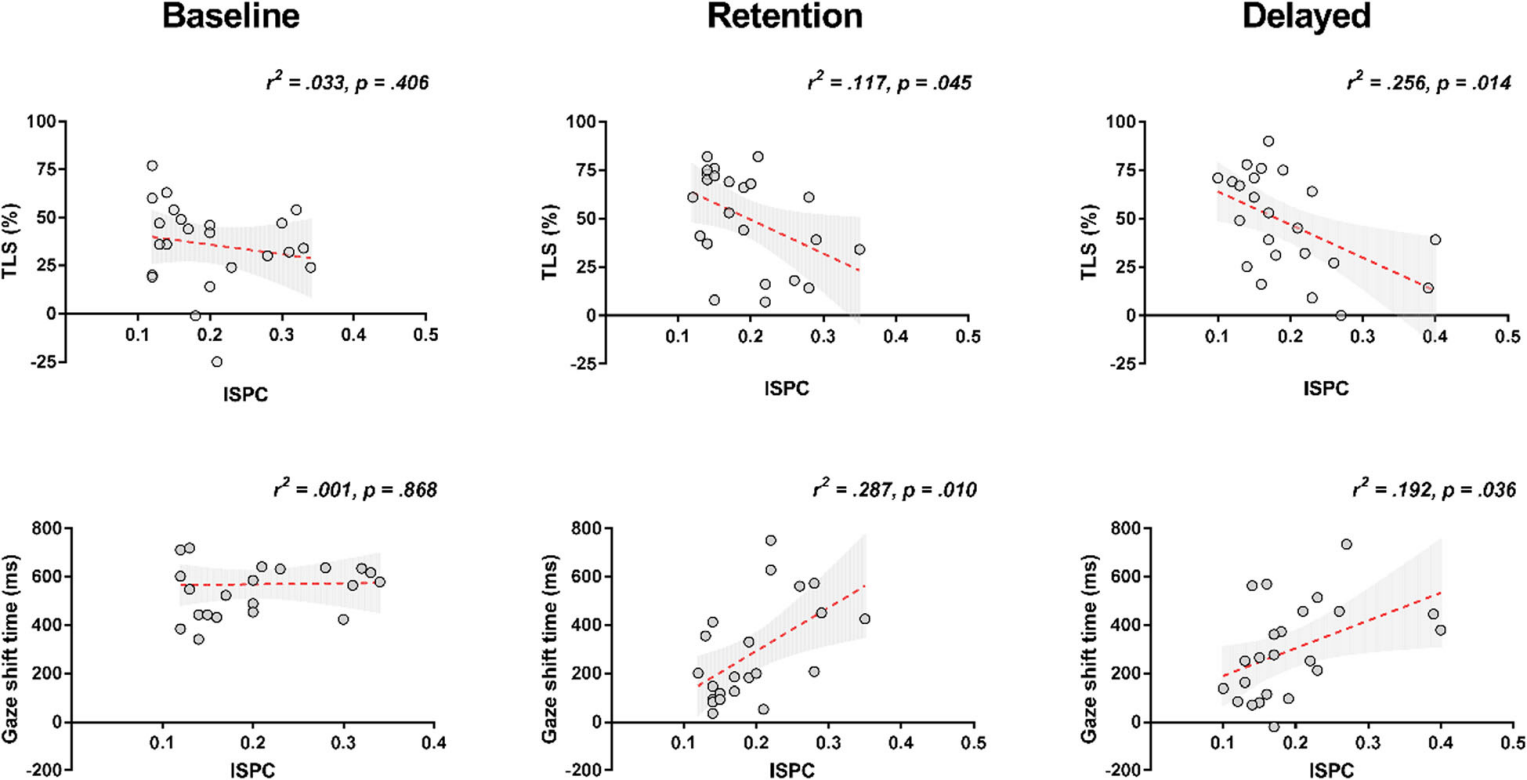
Relationship between gaze indices and conscious movement control Scatter plots displaying the relationship between TLS and T7-Fz (top row), and between gaze-shifting times and T7-Fz (bottom row), across three time points. Each plot displays the line of best fit (in red) with 95% confidence intervals (shaded in grey), the shared variance (*r*^2^) and the significance value (*p*) of each regression

At baseline, a non-significant regression equation was found when predicting T7-Fz connectivity based on TLS, *F* (1, 21) = 0.718, *p* = .406, *r*^*2*^ = .033, and gaze shifting, *F* (1, 21) = .028, *p* = .868, *r*^*2*^ = .001. At retention, however, both TLS, *F* (1, 21) = 4.532, *p* = .045, *r*^*2*^ = .177, and gaze shifting, *F* (1, 21) = 8.056, *p* = .010, *r*^*2*^ = .287, were significant predictors of T7-Fz connectivity. The same was true at delayed retention, with TLS, *F* (1, 21) = 7.238, *p* = .014, *r*^*2*^ = .256, and gaze shifting, *F* (1, 21) = 5.004, *p* = .036, *r*^*2*^ = .192, again significant predictors of T7-Fz connectivity (Fig. 6).

#### Transfer tea-making task

Due to time-locking synchronisation errors, EEG data for three participants could not be analysed for the tea-task.

#### Performance

Results showed no significant difference between the MT (*Mdn* = 73.20 s) and GT (*Mdn* = 64.55 s) groups’ performance time at retention (*H* (1) = 1.763, *p* = .184). There was also no difference between the MT (*Mdn* = 57.70) and GT (*Mdn* = 57.39 s) at delayed retention (*H* (1) = .033, *p* = .564).

#### Target locking score (TLS)

No significant main effect of time *F* (1, 22) = 3.799, *p* = .065, η_p_^2^ = .147, but a significant main effect of group*, F* (1, 22) = 22.328, *p* < .001, η_p_^2^ = .504, was observed, revealing participants in the GT group to exhibit significantly lower TLS compared to participants in the MT group. There was no significant time x group interaction, *F* (1, 22) = 0.009, *p* = .926, η_p_^2^ = 00.

#### Gaze shifting

Results revealed no main effect of time, *F* (1, 22) = 2.216, *p* = .151, η_p_^2^ = .092, no main effect of group, *F* (1, 22) = 3.151, *p* = .090, η_p_^2^ = .048, and no time x group interaction, *F* (1, 22) = 1.115, *p* = .302, η_p_^2^ = .048.

#### Alpha power

Results revealed no main effect of time, *F* (1, 18) = .257, *p* = .618, η_p_^2^ = .014, or group, *F* (1, 18) = .195, *p* = .664, η_p_^2^ = .011, but a main effect of ROI, *F* (3.495, 62.917) = 27.837, *p* < .001, η_p_^2^ = .607, revealing alpha power to be lowest over central and parietal regions, and highest overall temporal, frontal and occipital regions. All other interactions were non-significant.

#### High alpha connectivity

When examining cross hemispheric (T7 vs T8) changes in frontal (Fz) connectivity, results from ANOVA showed no significant main effect of time, *F* (1, 17) = 3.693, *p* = .072, η_p_^2^ = .178, or group, *F* (1, 17) = 3.248, *p* = .089, η_p_^2^ = .160. There was however a significant main effect of hemisphere, *F* (1, 17) = 11.694, *p* = .003, η_p_^2^ = .408, showing overall higher T7-Fz connectivity compared to T8-Fz connectivity.

#### Δ high alpha connectivity

**Fig. 7:**
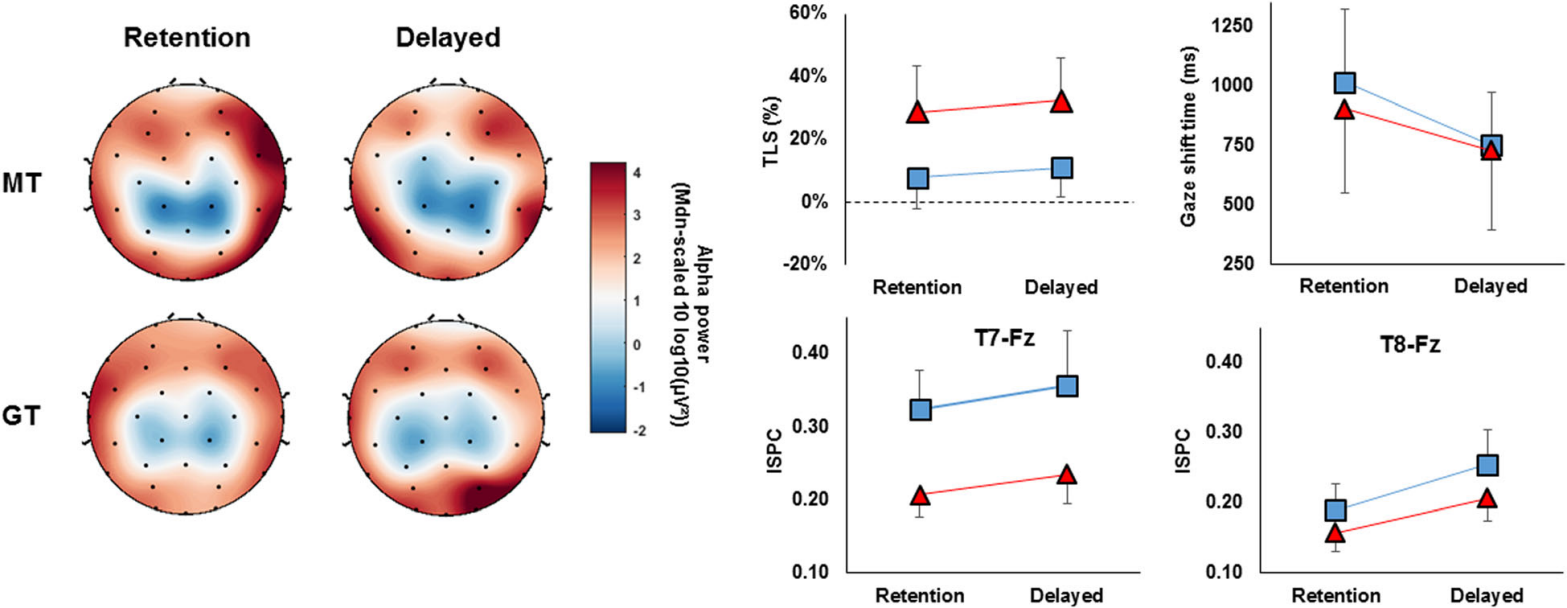
EEG data related to the transfer tea-making task Transfer tea-making task data showing scalp topographies representing regional alpha (left), mean (± SD) TLS and gaze shifting times (top-right) and mean (± s.e.m) T7-Fz and T8-Fz EEG connectivity (bottom-right) at both retention and delayed retention for both training groups

When examining Δ cross hemispheric (T7 vs T8) changes in frontal (Fz) connectivity, results from ANOVA showed no effect of time, *F* (1, 17) = 1.054, *p* = .318, η_p_^2^ = .055. There was however a main effect of hemisphere, *F* (1, 17) = 4.751, *p* = .041, η_p_^2^ = .232, and group, *F* (1, 17) = 4.977, *p* = .037, η_p_^2^ = .217, which was superseded by a significant hemisphere x group interaction, *F* (1, 17) = 4.751, *p* = .041, η_p_^2^ = .209. Follow up pairwise comparisons showed a significant difference between groups for T7-Fz connectivity (*p* = .022), in which the MT group exhibited a much greater increase from baseline compared to the GT group. The MT group also exhibited overall significant hemispheric asymmetry, with connectivity higher for T7-Fz compared to T8-Fz (*p* = .009), whereas the GT group did not (*p* = .906). Data for the transfer task can be seen in Fig. 7.

## Discussion

The aim of the second experiment was to determine the efficacy of GT in expediting prosthetic hand learning and alleviating the associated cognitive burden. We hypothesised that GT would optimise visual control, expedite skill acquisition, and promote neural efficiency by reducing conscious control, compared to MT instructions. We also hypothesised that these benefits would carry over to our complex transfer task [33]. Finally, we hypothesised that an increased dependence on vision to monitor the prosthesis would be related to increases in conscious movement control.

Supporting our hypothesis, results suggest that participants in the GT group implemented the training instructions by increasing their TLS and increasing the speed of their gaze shifts compared to the MT group (Fig. 4). Our results also show that by adopting more efficient gaze strategies, participants in the GT group performed consistently faster than the MT group from the first training session onwards. Although both groups exhibited a significant improvement in performance across time that somewhat plateaued by the third training session, the natural speed of participants in the GT group was ~ 20% faster than the MT group without being any more errorful. Encouragingly, the improved visual control adopted by the GT group also transferred to the more complex tea-making task, with participants using a higher TLS (~ 20%) compared to the MT group (Fig. 7).

While GT optimised gaze behaviour and expedited learning, we found mixed results when determining whether this decreased dependence on vision enhanced neural efficiency. For regional alpha power, we found a focal pattern consistent with Experiment 1, in which cognitive resources were primarily gated towards the central and parietal regions of the brain, regardless of training received. As such, our findings seemingly validate the utility of measuring regional alpha power to examine the functional architecture of the brain during prosthetic hand control. Although this gating pattern was insensitive to change from baseline to retention, there was a significant decrease in temporal alpha at delayed retention compared to baseline, regardless of which training was received. This increased excitability of the temporal regions is contrary to our predictions that increased skill would decrease (left) temporal activity. However, it should be noted that our predictions were primarily based upon research comparing expert-novice differences (as was seen in Experiment 1), or longitudinal training (~ 15 weeks) in target sports. Given the dynamic nature and complexity of our task, it is likely that the putative link between motor-skill expertise and optimal cortical organisation, as indexed by alpha *power*, might flexibly depend on external demands and required performance rather than a rigid strategy (always reduced activity [56];). Future research could explore this by conducting longitudinal intervention studies or by examining expert vs. novice comparisons of prosthesis users.

Our EEG results did however provide stronger evidence to suggest that GT reduces conscious verbal-analytical processes. Specifically, we showed that participants in the GT group exhibited a significant reduction in T7-Fz connectivity from baseline to retention and delayed retention, whereas the MT group did not (Fig. 5). We also showed a significant difference in the baseline change in T7-Fz connectivity between groups, with GT showing a decrease and the MT group showing an increase. The change in temporal-frontal connectivity also showed significant hemispheric asymmetry for the GT group, showing decreased T7-Fz and increased T8-Fz connectivity. Encouragingly, similar results were observed in the transfer tea-making task, with the training conditions again significantly altering the change in T7-Fz connectivity. However here, participants in the GT group displayed similar levels to that seen at baseline coin task performance, whereas the MT group showed a large increase.

These findings strongly suggest that encouraging learners to engage visual attention on the target rather than object manipulation, discourages burdensome verbal-analytical control [34]. In fact, regression analyses provided direct support for this claim, revealing that reduced T7-Fz connectivity was significantly predicted by increased TLS and faster gaze shifting times at retention and delayed retention. Conversely, our results also highlight how the provision of explicit instructions can accentuate the reliance on verbal processes, especially during complex tasks that are more reflective of the activities of daily living. Indeed, as these relationships were not present at baseline, the link between visual monitoring and conscious control appears to be highly dependent on the cognitive strategies encouraged through training rather than being inherent in prosthesis control. As conscious control processes require high cognitive demands they can result in performance breakdown under increasing task difficulty and fatigue [38] and should therefore be minimised in prosthesis rehabilitation.

These results provide evidence that GT alleviates conscious control and promotes neural efficiency, reducing the non-essential interaction between the motor planning and verbal-analytical regions of the brain. They also provide evidence that the provision of explicit instruction via MT can have the opposite effect, increasing the functional communication between motor-planning and verbal-analytical regions – an effect that increased during the more complex transfer task. Indeed, these findings are in line with previous research in laparoscopic surgery [49], and should not only act to promote the benefits of implicit learning via GT, but also act as a warning against the provision of more explicit training methods.

## General discussion

In this study, we report the first attempt to simultaneously examine the visuomotor and cortical mechanisms that contribute to the cognitive burden experienced by upper-limb prosthesis users [4, 13]. In both experiments, we provide further evidence that prosthetic hand control places high demands on visual attention and cognitive processes in order to guide and monitor movements, particularly during object manipulations [7]. Importantly, we also show that individuals can be trained to reduce their reliance on vision via GT, which subsequently expedites learning and encourages greater neural efficiency compared to more traditional explicit training methods. The findings of these experiments therefore have important theoretical and practical implications.

From a theoretical perspective, it is important to understand why prosthesis users appear to maintain these inefficient strategies despite skill improvements. In the development of eye-hand coordination, vision is initially utilised primarily as a feedback mechanism to monitor ongoing action as learners develop sensorimotor mapping rules between commands and movements, and between vision and proprioception [9]. However, for *typical* learners, as these mappings are refined, dependence of vision is relinquished from monitoring action and begins to be used as a feed-forward mechanism as soon as other senses (primarily touch and proprioception) can take over [8]. Prosthesis users’ over-reliance on visual feedback therefore represents a sensory substitution that is required to compensate for the severe deficits in proprioceptive and haptic feedback in this mapping process. Yet, considering the ease at which participants were trained to stop looking at the prosthesis in Experiment 2, it is clear that this strategy is not efficient nor a prerequisite of successful prosthesis control. So how then does GT help to overcome such deficits? And why might this be beneficial to long-term prosthesis control?

There are a number of potential theoretical explanations for this. First, being trained to use vision in this more proactive manner and to “*look at the right place at the right time”* is thought to aid effective coordination of the visuomotor system [8, 9, 43]. Specifically, by adopting early and accurate look-ahead fixations users are able to effectively pass visually acquired target-related information to the motor system so accurate movements can be planned and executed [8, 9]. The faster performance times exhibited by the GT group support these predictions, and suggest increased proficiency of movements. Second, reducing the dependence on vision reduces conscious movement control, supporting the idea that GT alleviates the reliance on these explicit and burdensome processes [34]. Third, it could be that case that GT forces the development of ‘new’ sensorimotor mapping rules using the remaining senses (e.g., proprioception, or auditory information from the prosthesis’ motors [13]) to enable vision to be used in a more proactive feed-forward manner.^2^ Finally, the benefits of GT could also be attributable to encouraging learners to adopt an external focus of attention (FOA). Research has shown that focusing on the effect of movement (external FOA) rather than the mechanics of the movement itself (internal FOA) promotes better performance in a variety of movement contexts [57]. Interestingly, an external FOA has also been shown to improve movement economy by reducing muscle stiffness and activity [58]. Reducing demands on muscle fibre recruitment may therefore mitigate the negative effects of fatigue upon electromyographic (EMG) signal quality [58] and improve long-term myoelectric control.

From an applied perspective, the methods used in these experiments could be used to assess the usability of prosthetic hands from a design perspective. While the technological development of hand prosthesis is increasing rapidly, research examining the usability and interaction between the user and the prosthesis is lacking. For example, while performance measurements are adequate in accessing the functionality of prosthesis hand devices, they are not sensitive enough to assess their usability. As our transfer task shows, both training groups performed similarly but the magnitude of mental resources needed to perform was significantly less in the GT group. So, just because a user can *use* a hand prosthesis does not mean that the hand prosthesis is intuitively *useable*. From technologies that provide vibrotactile feedback [59] to hands that can actually ‘see’ for themselves [60], each will increase or lessen the cognitive resources needed to interact with the world. It is this user-prosthesis-world interaction that needs examining in future research, which to be effective, will depend on significant collaborations between applied psychologists, prosthesis engineers, occupational therapists and prosthesis users themselves.

Similarly, an examination of the cognitive demand experienced during prosthesis learning could also aid occupational therapists to assess a patient’s progress. However, the methods used in these studies are probably not cost effective given the expensive equipment required and the expertise needed to operate it. Researchers therefore should develop and validate a multidimensional workload measure specific to prosthesis use. Such a measure has previously been developed for surgical skills (SURG-TLX; [61], and would allow for more cost-effective and immediate clinical assessment of the cognitive demand experienced by prosthesis users during the rehabilitation process.

Despite the important first steps presented here, several limitations should be noted. First, we are limited by our use of intact users of a simulator rather than patients with limb loss. However, evidence has shown that these populations display comparable kinematic profiles [1], visuomotor behaviours [6, 7], and perceptual experiences [62], suggesting that using a simulator provides a useful surrogate to examine the sensory-motor deficits that prosthesis users face. Yet, it is unclear how increasing the length of the operating arm when using the prosthesis simulator (approximately 7 cm when the hand is unclenched) independently influences visuomotor and neurophysiological behaviours. Furthermore, the cortical reorganisation that occurs following amputation can cause large-scale changes in neural networks, making direct transfer of our results to an amputee population potentially difficult. For example, evidence shows that neuroplasticity of the cortex following amputation can promote an expansion of the residual limb segments into the former limb territory [63], and promote a progressive disconnection of the missing hand cortex and the sensorimotor cortex [64]. Clearly, future work is needed to evaluate the cognitive burden in a clinical population and to explore if this can be alleviated in the same manner using a GT intervention.

The degree of ambiguity in the temporal accuracy of EEG data must also be highlighted. Here, data were segmented through clearly defined epoch lengths relative to a given manual action (i.e., jar lift in experiment 1). Whilst this method enabled meaningful comparisons to be made, it fails to guarantee that the segmented data represent the exact same “portion” of movement on a trial-to-trial basis. Though unfavourable, this inaccuracy appears a necessary compromise for investigating EEG during dynamic motor tasks, that should be addressed in future research.

Finally, in these studies we limited our EEG analysis to the alpha frequency band in order to contextualise our findings with previous research on alpha gating [31] and connectivity during movement execution [20, 49]. In future, more exploratory research could benefit from investigating multi-scale interactions across different frequencies in order to acknowledge the fact that changes in specific frequency bands do not occur in isolation [65]. Such analyses could help to attain a more holistic understanding of the cortical disruptions evident during initial hand use and this could help develop objective methods to assess training programmes in the future.

## Conclusions

We believe that these two experiments represent the most comprehensive evaluation of the visual and cortical mechanisms relating to the cognitive burden associated to prosthetic hand control. We also demonstrate the efficacy of a GT intervention designed to alleviate this burden. This is important because this intervention seems to promote better learning and transfer, increased neural efficiency and both of these factors are what prosthesis users actually desire in a functional prosthetic hand [4]. This demonstrates that the problem of making prosthesis hands more useable is not necessarily a technological issue – both groups used the same hand in our study – but an issue relating to *how* the user interacts with this technology. Therefore, in future research and development we propose that a greater emphasis should be placed on understanding human factors alongside technological ones.

## Additional files

Additional file 1: A breakdown of the 17 task phases and 16 AOIs for the tea-making transfer task. (PDF 998 kb)

Additional file 2: Results from the additional “white noise” condition administered during Experiment 2. (PDF 589 kb)

## Abbreviations

ANOVA: Analysis of variance
AOI: Area of interest
ASL: Applied Science Laboratories
CMS: Common mode sense
DRL: Driven right leg
EEG: Electroencephalography
EMG: Electromyographic
EOG: Electrooculographic
FFT: Fast Fourier Transform
FOA: Focus of attention
GT: Gaze training
HEOG: Horizontal electrooculogram
IAF: Individual alpha frequency
ISPC: Inter site phase clustering
MT: Movement training
ROI: Region of interest
SHAP: Southampton Hand Assessment Procedure
TLS: Target locking strateg
VEOG: Vertical electrooculogram

## Footnotes

1 The sampling rate of the eye-tracker was 30 Hz, providing temporal accuracy of approximately ±33 ms when determining the onset of the *Lift* phase. Although this level of precision may not be suitable to detect specific event-related changes in EEG activity, it is not necessary for the present investigation that instead examines the consistency of spectral power across the entirety of a long time-window (1500 ms).

2 We did explore this explanation in an additional condition that eliminated auditory feedback via in-ear white noise. This had no effect on performance, gaze or neural activity after training (see Additional file 2).
