# Supplementary material for: Visual attention, EEG alpha power and T7-Fz connectivity are implicated in prosthetic hand control and can be optimized through gaze training
Source: J Neuroeng Rehabil. 2019 Apr 27;16:52. doi: 10.1186/s12984-019-0524-x (PMC6487034; doi:10.1186/s12984-019-0524-x)
Supplement: Supplementary file 1 — A breakdown of the 17 task phases and 16 AOIs for the tea-making transfer task. (PDF 998 kb) [file 12984_2019_524_MOESM1_ESM.pdf]

Additional File 1: Transfer tea-making task gaze behaviour analyses

Table S1. Details of the 17 task phases identified for our transfer task, with each phase onset and offset indicated.

#	*Task phase*	*Onset*	*Offset*	*Screenshot*
1	Mug transport	Mug grasped	Mug released	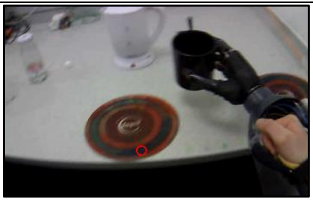
2	Teabag reach	Release of previous object	Teabag grasped	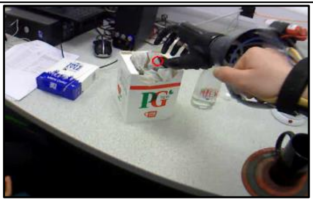
3	Teabag transport	Teabag grasped	Teabag released	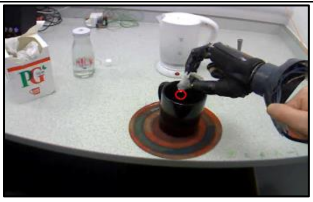
4	Kettle reach	Release of previous object	Kettle grasped	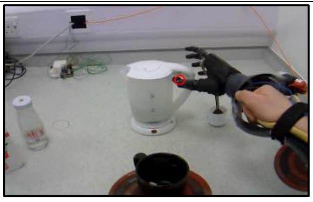
5	Kettle transport	Kettle grasped	Pour initiated	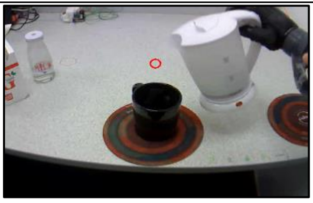
6	Kettle pour	Pour initiated	Pour finished	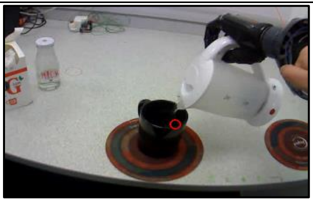
7	Kettle return	Pour finished	Kettle released	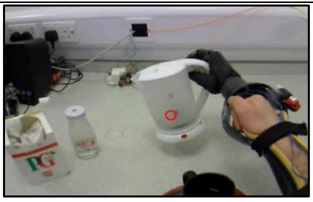

8	Sugar reach	Release of previous object	Sugar grasped	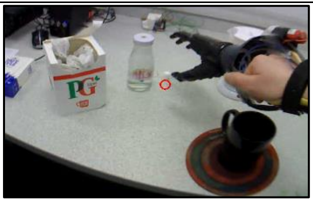
9	Sugar transport	Sugar grasped	Sugar released	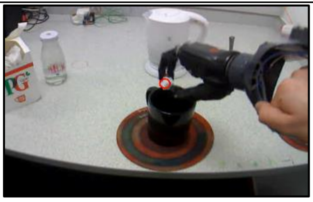
10	Milk reach	Release of previous object	Milk grasped	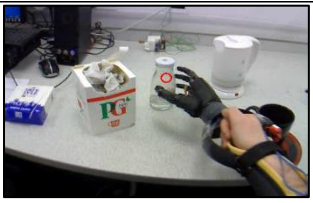
11	Milk transport	Milk grasped	Pour initiated	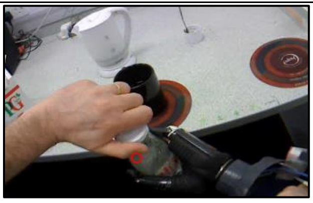
12	Milk pour	Pour initiated	Pour finished	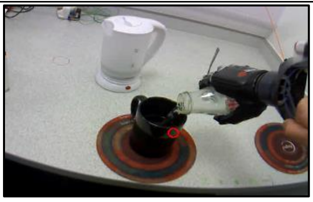
13	Milk return	Pour finished	Milk released	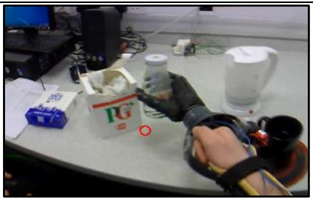
14	Spoon reach	Release of previous object	Spoon grasped	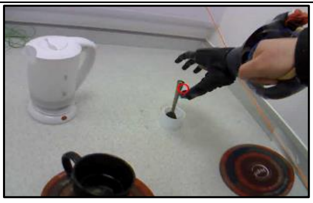
15	Spoon transport	Spoon grasped	Spoon emerged in tea	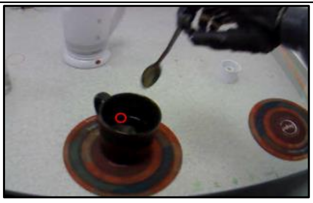

16	Spoon stir	Spoon submerged in tea	Spoon emerged from tea	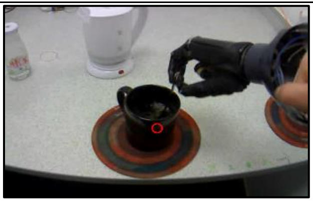
17	Spoon return	Spoon emerged from tea	Spoon released	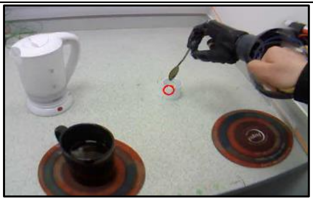

Table S2. Screenshots with all areas of interest highlighted in yellow.

Area of interest (AOI)	***Screenshot***
Mug	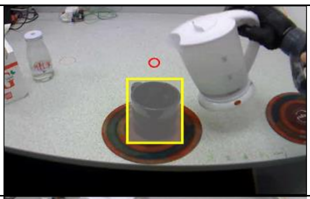
Prosthesis	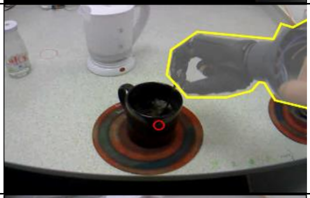
Place mat	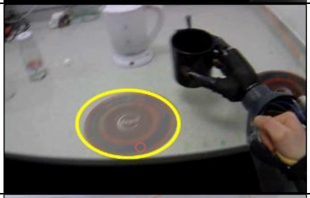
Kettle GCA	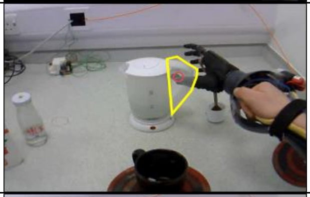
Kettle upper	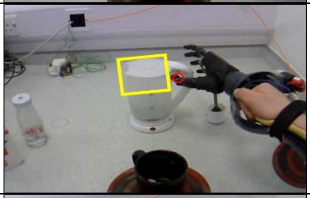
Kettle lower	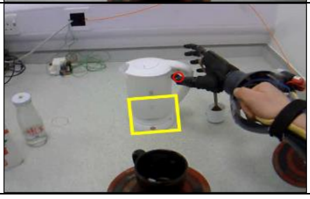

Kettle home	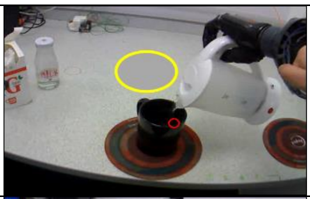
Teabag box	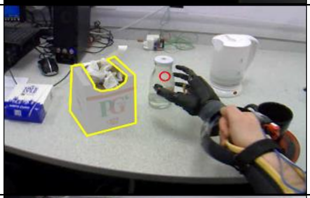
Teabag	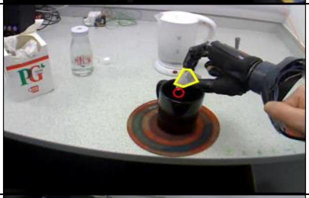
Sugar cube	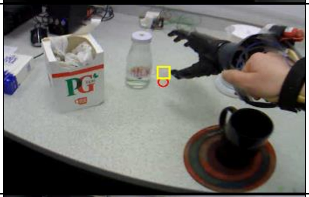
Milk GCA	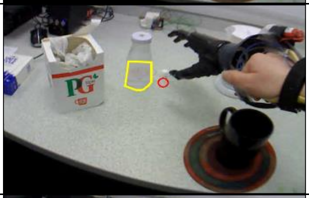
Milk upper	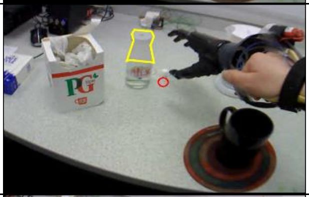
Milk home	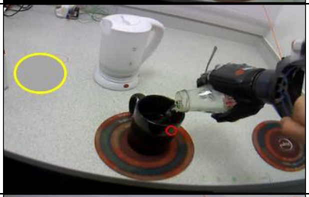
Spoon GCA	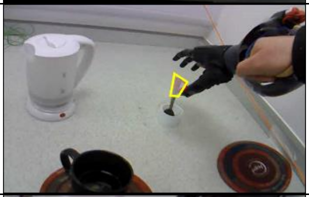
Spoon lower	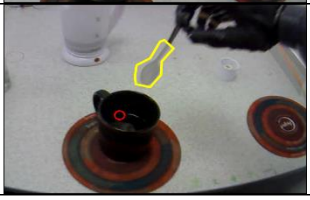

Spoon home	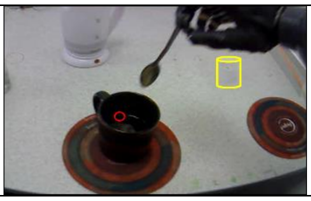
