# Supplementary material for: Visual attention, EEG alpha power and T7-Fz connectivity are implicated in prosthetic hand control and can be optimized through gaze training
Source: J Neuroeng Rehabil. 2019 Apr 27;16:52. doi: 10.1186/s12984-019-0524-x (PMC6487034; doi:10.1186/s12984-019-0524-x)
Supplement: Supplementary file 2 — Results from the additional “white noise” condition administered during Experiment 2. (PDF 589 kb) [file 12984_2019_524_MOESM2_ESM.pdf]

Additional File 2: Exploring the role of auditory feedback in the development of predictive prosthesis control.

Methods

In an exploratory investigation, an additional condition was included in Experiment 2 to determine the importance of auditory feedback in development of prosthesis control. To do so, we included an additional 15 trials of our coin task at the retention and delayed retention visits. However, for this block of trials, auditory feedback (from the prosthetic hand motors) relating the grasping action was eliminated for each participant by inserting in-ear headphones (Apple Inc., United States) that played 'white noise' during the coin task. To ensure auditory feedback was eliminated, participants were asked to continually open and close the prosthesis whilst the researcher slowly increased the volume. Once participants were sure they could no longer hear the prosthesis they signalled the researcher and the volume was set.

All equipment and analyses were performed according to the methods presented in the primary experiment.

Statistical analyses

All performance and gaze measures were subject to separate 2 (group; GT vs. MT) x 2 (time; retention & delayed retention) x 2 (noise; no noise & white noise) repeated measures analysis of variance (RM-ANOVA). Regional EEG alpha power was examined via a 2 (group) x 2 (time) x 2 (noise) x 7 (region) RM-ANOVA. EEG coherence was examined via a 2 (group) x 2 (time) x 2 (noise) x 2 (hemisphere) RM-ANOVA.

Results

Performance time. Results revealed no effect of noise, $F(1, 22) = 0.148$, $p = .704$, $\eta_p^2 = .007$, and no noise x group, $F(1, 22) = 0.390$, $p = .539$, $\eta_p^2 = .017$, noise x time, $F(1, 22) = 0.026$, $p = .873$, $\eta_p^2 = .001$, or noise x time x group, $F(1, 22) = 0.032$, $p = .859$, $\eta_p^2 = .001$, interactions.

Performance error. Results revealed no effect of noise, $F(1, 22) = 0.330$, $p = .572$, $\eta_p^2 = .015$, and no noise x group, $F(1, 22) = 0.111$, $p = .743$, $\eta_p^2 = .005$, noise x time, $F(1, 22) = 3.005$, $p = .097$, $\eta_p^2 = .015$, or noise x time x group, $F(1, 22) = 0.621$, $p = .439$, $\eta_p^2 = .027$, interactions.

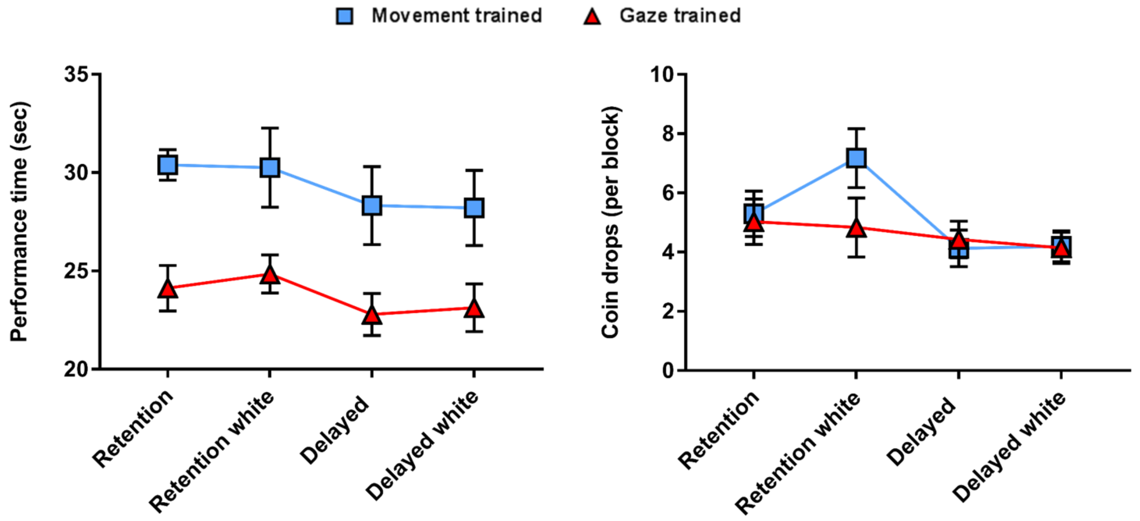

Figure 1. Mean (± s.e.m) performance times (left) and performance error (right) for both the MT and GT groups recorded at retention and delayed retention, both with and without white noise. In this instance, error is reflected by the number of dropped coins within each block of 15 trials (total 75 coins).

Target locking score (TLS). Results revealed no effect of noise, $F(1, 22) = 0.009$, $p = .927$, $\eta_p^2 = .000$, and no noise x group, $F(1, 22) = 0.195$, $p = .662$, $\eta_p^2 = .009$, noise x time, $F(1, 22) = 0.020$, $p = .889$, $\eta_p^2 = .001$, or noise x time x group, $F(1, 22) = 2.536$, $p = .126$, $\eta_p^2 = .103$, interactions.

Gaze shifting. Results revealed no effect of noise, $F(1, 22) = 0.695$, $p = .961$, $\eta_p^2 = .000$, and no noise x group, $F(1, 22) = 0.695$, $p = .695$, $\eta_p^2 = .031$, noise x time, $F(1, 22) = 0.964$, $p = .337$, $\eta_p^2 = .042$, or noise x time x group, $F(1, 22) = 0.497$, $p = .488$, $\eta_p^2 = .022$, interactions.

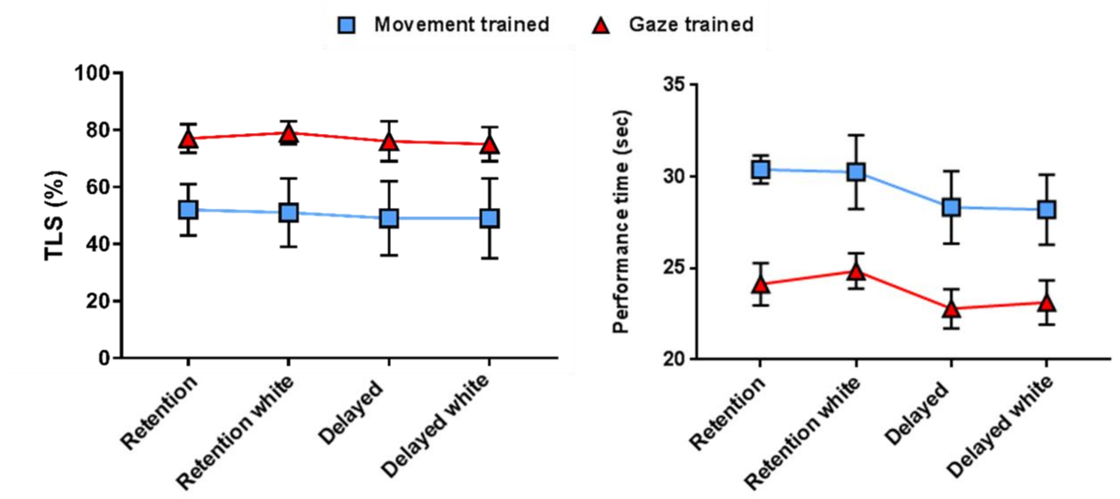

Figure 2. Mean (± SD) TLS (left) and gaze shifting (right) times for both groups, at retention and delayed retention, with and without white noise.

EEG alpha power. For the grasp phase, results showed no overall main effect of noise, $F(1, 21) = 1.423$, $p = .247$, $\eta_p^2 = .066$. There were also no significant interactions. Similarly, for the lift phase results showed no main effect of noise, $F(1, 21) = 0.113$, $p = .740$, $\eta_p^2 = .006$. All interactions were also non-significant.

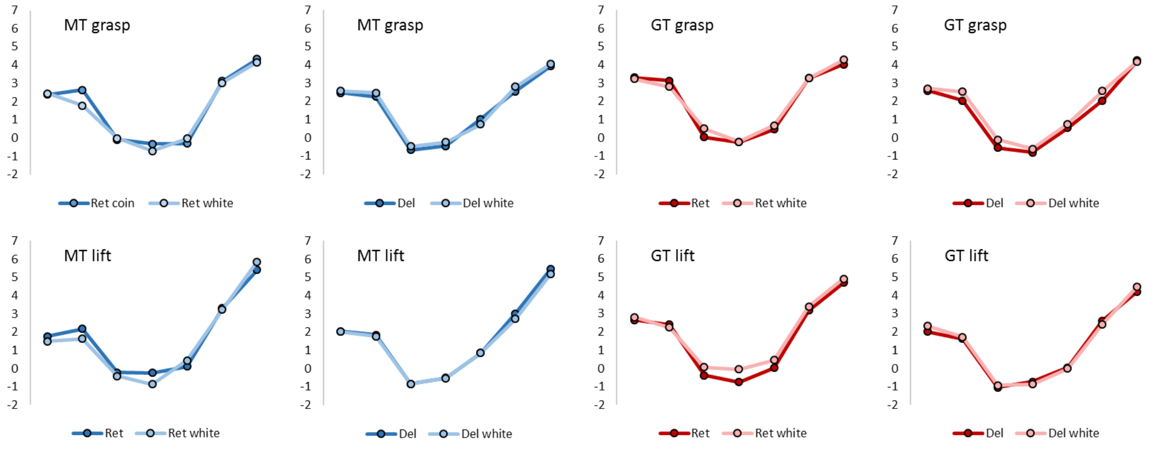

Figure 3. Collection of line plots displaying the regional alpha power for each group (MT & GT), each task phase (grasp & lift), each time point (Retention and Delayed), both with and without white noise. For plotting brevity, the Y axis for all plots reflects Alpha power (Mdn-scaled 10*log10(μV²)),

and the X axis reflects seven distinct brain regions in the following left to right order: left temporal, frontal, left central, right central, parietal, right temporal, occipital.

EEG coherence. For the grasp phase results showed no main effect of noise, $F(1, 21) = 0.180$, $p = .676$, $\eta_p^2 = .009$. All further interactions were non-significant. For the lift phase results showed no main effect of noise, $F(1, 21) = 0.550$, $p = .472$, $\eta_p^2 = .027$. All further interactions were non-significant.

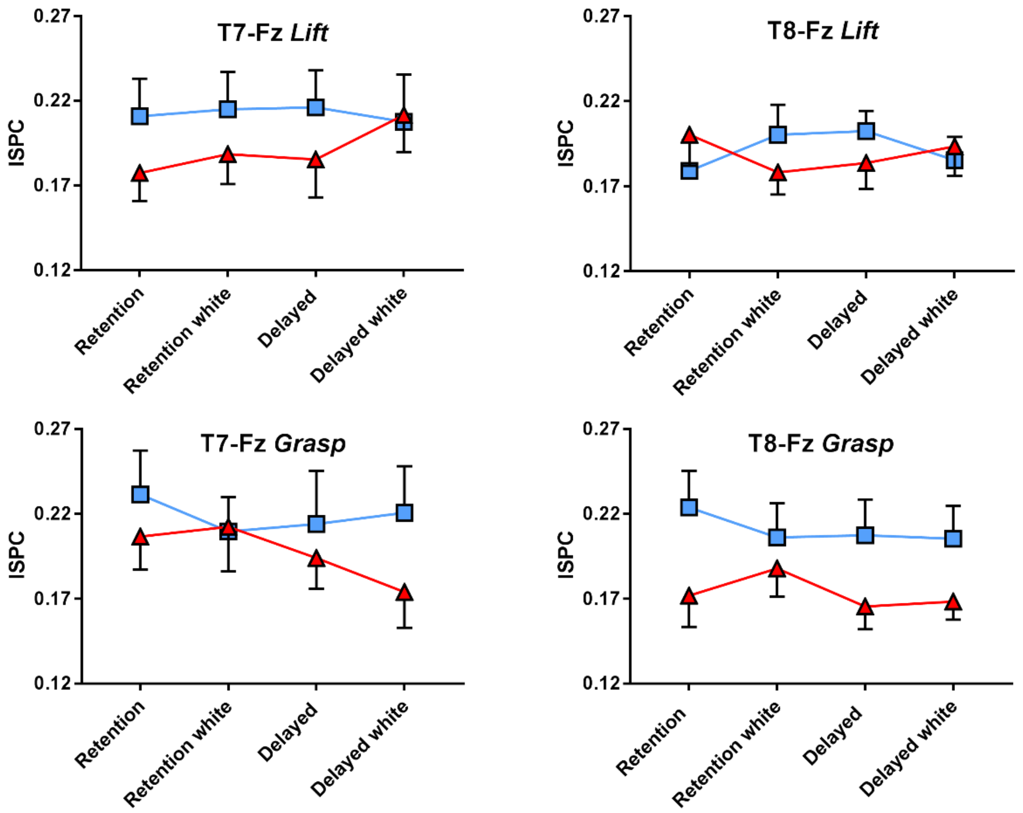

Figure 4. Line plots displaying mean (± s.e.m) EEG coherence calculated via inter-site phase clustering (ISPC) over time. Values for each group, each time point, and each condition, are displayed separately for T7-Fz (left column) and T8-Fz (right column) coherence, and for the Lift (top row) and Grasp (bottom row) task phases.
